# Rapid in situ ^13^C tracing of sucrose utilization in Arabidopsis sink and source leaves

**DOI:** 10.1186/s13007-017-0239-6

**Published:** 2017-10-18

**Authors:** Frederik Dethloff, Isabel Orf, Joachim Kopka

**Affiliations:** 1 Department of Translational Research in Psychiatry, Max Planck Institute of Psychiatry, Kraepelinstr. 2-10, 80804 Munich, Germany; 2 French Associates Institute for Agriculture and Biotechnology of Drylands, The Jacob Blaustein Institutes for Desert Research, Ben-Gurion University of the Negev, Sede Boqer, Israel; 3 Max Planck Institute of Molecular Plant Physiology, Am Mühlenberg 1, 14476 Potsdam-Golm, Germany

## Abstract

**Background:**

Conventional metabolomics approaches face the problem of hidden metabolic phenotypes where only fluxes are altered but pool sizes stay constant. Metabolic flux experiments are used to detect such hidden flux phenotypes. These experiments are, however, time consuming, may be cost intensive, and involve specialists for modeling. We fill the gap between conventional metabolomics and flux modeling. We present rapid stable isotope tracing assays and analysis strategies of ^13^C labeling data. For this purpose, we combine the conventional metabolomics approach that detects significant relative changes of metabolite pool sizes with analyses of differential utilization of ^13^C labeled carbon. As a test case, we use uniformly labeled ^13^C-sucrose.

**Results:**

We present petiole and hypocotyl feeding assays for the rapid in situ feeding (≤ 4 h) of isotopically labeled metabolic precursor to whole *Arabidopsis thaliana* rosettes. The assays are assessed by conventional gas chromatography–mass spectrometry based metabolite profiling that was extended by joined differential analysis of ^13^C-labeled sub-pools and of ^13^C enrichment of metabolites relative to the enrichment of ^13^C-sucrose within each sample. We apply these analyses to the sink to source transition continuum of leaves from single *A. thaliana* rosettes and characterize the associated relative changes of metabolite pools, as well as previously hidden changes of sucrose-derived carbon partitioning. We compared the contribution of sucrose as a carbon source in predominantly sink to predominantly source leaves and identified a set of primary metabolites with differential carbon utilization during sink to source transition.

**Conclusion:**

The presented feeding assays and data evaluation strategies represent a rapid and easy-to-use tool box for enhanced metabolomics studies that combine differential pool size analysis with screening for differential carbon utilization from defined stable isotope labeled metabolic precursors.

**Electronic supplementary material:**

The online version of this article (doi:10.1186/s13007-017-0239-6) contains supplementary material, which is available to authorized users.

## Background

Plant carbon metabolism has been studied for several decades using radioactive and stable carbon isotopes. Since the development of high throughput mass spectrometry (MS) methods for metabolomics approaches, research of plant metabolism has made great progress [1]. The most common methods in plant metabolomics are liquid chromatography–MS (LC–MS) or gas chromatography–MS (GC–MS). Metabolomics approaches usually provide only a snapshot of metabolism, more precisely of the current metabolite pool sizes which are assumed to be in a steady state. Carbon fluxes between the single metabolites cannot be assessed by classical metabolomics approaches, and even the prediction of fluxes using relative or absolute changes of pool sizes is rarely feasible. Many studies aim to overcome this problem by analyzing time series to monitor the transient changes of metabolites, e.g., [2, 3]. However, there are several examples of changes in flux rates without noticeable changes of corresponding metabolite pool sizes [4, 5]. One of the most fundamental examples was reported by Stitt [4]. In this case strong changes in the rate of photosynthesis did not lead to notable changes in pool sizes of Calvin–Benson–Bassham cycle intermediates.

The investigation of metabolic fluxes requires experimental approaches that differ from conventional metabolomics experiments. Tracing of radioactively or stably labeled isotopes are commonly performed to address this problem. The first experimental challenge of labeling experiments is to provide the labeled compound/element to the plant, organ, tissue, or cell, where the pathway of interest is located, while ensuring a minimum perturbation of the system. The most elegant method used in plant research on metabolic fluxes is the tracing of carbon isotopes using photosynthetic labeling. The perturbation of the plant is by far the lowest possible when using labeled CO_2_ as an entry point into the metabolism, e.g., [6].

Major achievements in understanding the principles of plant life underpin the importance of labeling experiments: both the Calvin–Benson–Bassham cycle and photorespiration in plants were elucidated by photosynthetic labeling [7, 8]. The carbon that is fixed during photosynthesis is stored in sucrose and starch [9], which are located in the vacuole [10–12] and chloroplast [13], respectively. Carbon transport occurs [14] mainly in the form of sucrose transport from source tissues to sink tissues like flowers, seeds, roots, and young leaves [15, 16]. During vegetative growth the young leaves are the predominant sink tissue but they maturate during their development and become source leaves. This transition from predominantly sink to predominantly source leaf function, i.e. from net carbon importer to net exporter, occurs between 30 and 60% of final leave size [17, 18].

With the above mentioned development of high throughput metabolomics approaches, isotope tracing has gained new attention in systems biology for estimating fluxes through specific pathways in the metabolic network or even the modeling of whole cell metabolic fluxes [19, 20]. Measurement and interpretation of flux data is time consuming and cost intensive work that demands complex data analysis and specialized laboratories. The compartmentation of plant cells imposes another level of complexity and experimental challenge on fluxomics [21]. To overcome this problem partial labeling strategies with position specific labeled metabolic precursors have been developed [22]. These position specific labeled metabolic precursors have been verified and optimized by bioinformatics modeling approaches to detect fluxes of specific pathways [23, 24].

In order to determine in vivo fluxes, kinetic labeling is used, i.e. samples are labeled for various time intervals [25], or plants are fully photosynthetically labeled and the chase, i.e. the fading of the label out of the metabolism, is measured at different points in time [6]. Most recently, measurements have been combined with modeling approaches to deduce global flux information from cell culture [26] and even in whole *A. thaliana* labeling experiments [27].

Besides photosynthetic labeling, different feeding approaches have been reported for labeling plants [28–30]. The majority of approaches followed classical approaches like leaf disc assays [25, 31], petiole feeding assays with single detached leaves [32], or whole seedling assays in liquid culture [24, 26, 33].

Most of the investigations were focused on specific pathways or a targeted question [28–33] and were not aiming for a non-targeted screening approach. Samples were typically pooled for the analyses so that information on individual plants, rosettes, or leaves was lost. And more importantly, a general feeding assay for diverse isotope labeled metabolic precursors and easy-to-use analysis is still missing for non-photosynthetic labeling approaches.

Here, we introduce simple feeding assays for the *A. thaliana* model plant that aim to capture flux snapshots of multiple leaves from the same rosette and present analysis methods that maintain the metabolomics screening character and thus add on to metabolomics studies using LC- or GC–MS methods for targeted and non-targeted analyses of altered metabolic fluxes. The feeding assays are designed in a way that a range of different labeled metabolic precursors can be entered into the assay and fed to an *A. thaliana* rosette with little additional optimization. By feeding uniformly labeled ^13^C-sucrose we demonstrate the feasibility of our feeding assay and subsequent data analyses. Our exemplary experiments estimate the distribution of carbon from sucrose into central carbon metabolism. We show that carbon from sucrose is utilized differently at the progressive developmental stages of multiple leaves from a single *A. thaliana* rosette. Besides metabolic pool size characterization of these different leaf stages, carbon tracing provides additional information about the direction of a general carbon flow in a given pathway. In addition we show how to easily detect possible candidate metabolites with altered carbon partitioning and flux regulation. With this technology we fill the gap between classical metabolomics and complex fluxomics modeling with a rapid combined screening for altered metabolite pools and carbon utilization.

## Results

Our method developments focus on the *A. thaliana* rosette, which is still one of the most common model systems in plant science. The *A. thaliana* rosette might look homogeneous but, in fact, is a complex system that comprises leaves in different developmental stages. We investigated this system as a “proof of concept” study and demonstrate that metabolism can be indeed quite different in single leaves across an *A. thaliana* rosette. We demonstrate that dissecting rosettes into single leaves or groups of leaves at similar developmental stages will generate more detailed physiological insights than the frequently used pools of whole rosettes. In the following, we will first introduce our test system and demonstrate the developmental reprogramming of metabolism across the single leaf stages within the *A. thaliana* rosette. We will then describe in detail two experimental approaches for the isotope labeling of whole rosettes and finally discuss respective results and the method’s potential for enhanced physiological studies.

### Physiological staging of *A. thaliana* leaves for reproducible metabolomics studies

We selected the vegetative growth stage to study the developmental reprogramming of metabolism within the rosette system of *A. thaliana* Col-0. Under 8 h short day conditions, populations of *A. thaliana* Col-0 reached developmental stage 1.12–1.13 [34] with 12.6 ± 1.5 leaves (average ± standard deviation, n = 14) at 36 days after imbibition. The vegetative development of plants used in this study was limited to 1 week before the appearance of the first floral bud in the center of the rosette. The cotyledons and the first 3–4 juvenile leaves [35] within the rosette were already partially or fully covered by adult leaves in most plant individuals. The resulting high variability in shading of the juvenile leaves at this stage caused us to exclude these leaves from further analysis.

To ensure the lowest possible variance between biological replicates in our experiment we assessed the progression of developmental stages of sampled leaves at whole plant stage 1.12–1.13. For this purpose we tested different measures of non-invasive ontogenic staging of *A. thaliana* rosette leaves. The conventional ontogenic staging system of *A. thaliana* leaves starts with the chronologically oldest juvenile rosette leaf and progresses to successively younger adult leaves. The application of this staging system to our plant populations and rosette system did not yield uniform anatomical and developmental leaf stages, because the plant populations could only be synchronized to ± 1.5 leaves across the plant individuals of our populations. Alternatively, the developmental stages of single leaves can be characterized by percentage of leaf area relative to the final maximal area that is reached by each leaf [36]. Staging by relative leaf size is the optimal choice for analyzing leaf development in non-invasive studies. But this approach was obviously not applicable due to the requirement for destructive sampling.

**Fig. 1:**
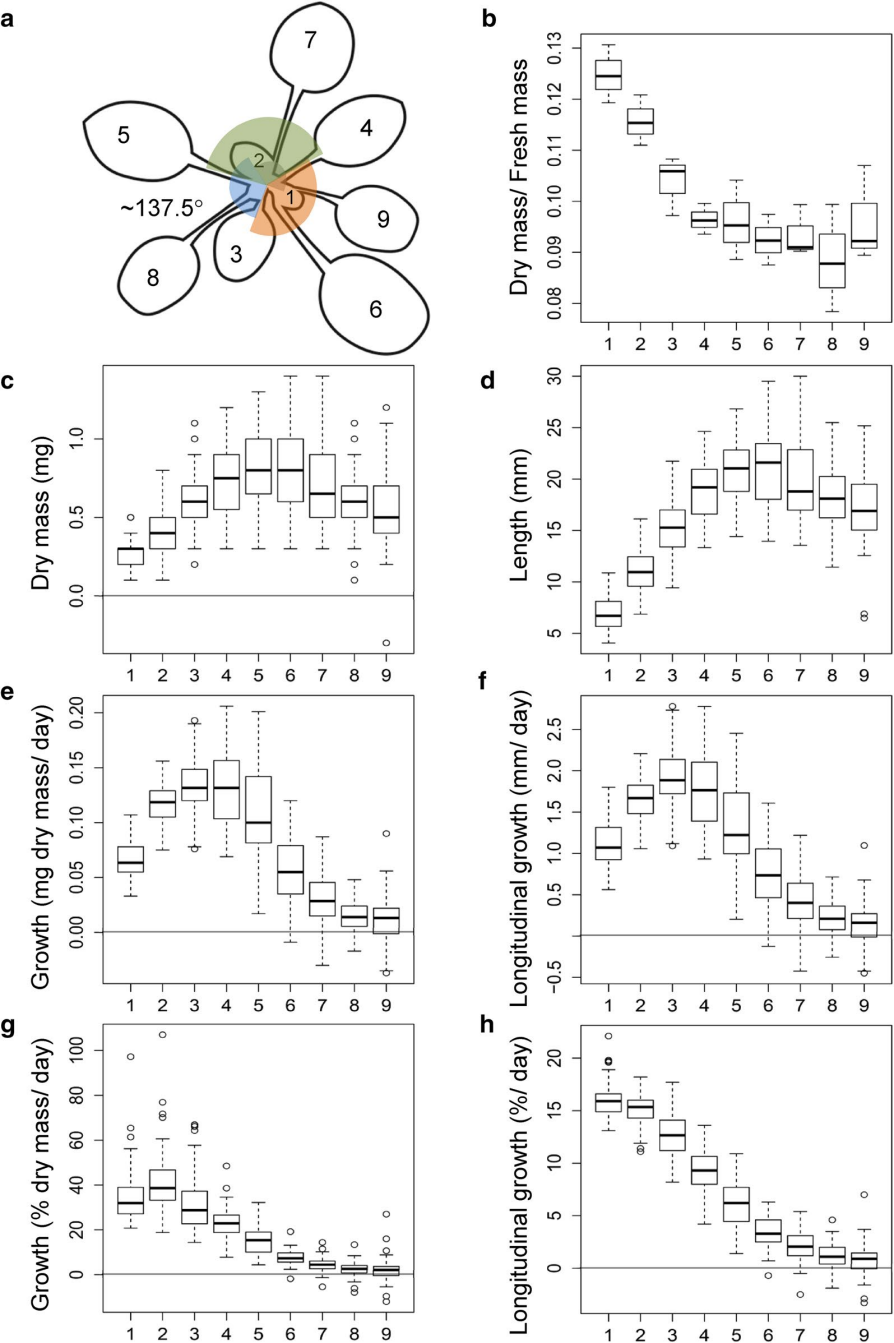
Growth characteristics of leaf positions P1–P9 of an *A. thaliana* Col-0 rosette in 8 h short days at vegetative developmental stage 1.12–1.13. **a** Scheme of leaf positions P1–P9. P1 is the youngest fully visible leaf. P1 has on average 7.1 mm length and 0.29 mg dry mass. Older leaves are shifted in the majority of individuals counter-clock wise by angles of 137.5°, respectively. Juvenile leaves and cotyledons are not shown, **b** dry mass to fresh mass ratio, average of 3 independent pools of 20 leaves, **c**, **d** dry mass and leaf length, **e**, **f** gain of dry mass per day and longitudinal growth, **g**, **h** relative gain of dry mass and length compared to leaf measurements at stage 1.12–1.13 (median and box-plot of n = 68 plants)

To obtain leaf stages of approximately equal anatomical and developmental properties we used a staging system that started with the chronologically youngest leaf in the center of the rosette, i.e. leaf position P1 with a visible lamina of < 5 mm length (Fig. 1a; Additional file 1: Table S1). The positions P2–P9 were defined to represent the successively older leaves. The ratio of dry mass to fresh mass classified leaf positions P1–P3 into the phase of rapid expansion growth and water uptake. Later stages had a constant dry to fresh mass ratio (Fig. 1b). Leaf length and calculated dry mass showed that rapid growth continued up to position P5 (Fig. 1c, d). In agreement with the expected changes of growth potential, positions P6–P9 had increasingly larger sizes at whole plant stage 1.12–1.13. Growth rates accelerated to a maximum at P3 and approximated a minimal but non-zero rate at P7–P9 (Fig. 1e, f). The relative gain of dry mass and length decreased continuously from position P2 to P9 (Fig. 1g, h).

The growth characteristics of the leaf staging system used in this study was matched to a generalized qualitative model of leaf growth [36]. In this model the percentage of final leaf area was used to aggregate information on anatomical, developmental, and physiological processes that occur during leaf ontogeny. The changes of growth rate, dry mass accumulation, and relative leaf expansion rate, which were included in this model, allowed the match of our staging system to this model. Our data placed positions P1–P3 into the sink phase of leaf development. A maximum carbon import into the leaf is expected at P3. Position P4 matched to the transition stage with an approximately neutral carbon import to carbon export balance. Positions P5 to P9 were classified as source phase leaves, for which a net export of carbon into the rosette is expected. Senescent leaves were not observed at whole plant stage 1.12–1.13 under our cultivation conditions [37].

### Conventional metabolomics profiling reveals a gradual change of the metabolic phenotype during sink to source transition of the *A. thaliana* leaf

For the purpose of finding characteristic marker metabolites and metabolic patterns that are associated with leaf growth and development, we performed conventional metabolomics without liquid phase partitioning that monitors both small polar and small lipophilic compounds, as described previously by method variant 3.2.3 of [38].

**Fig. 2:**
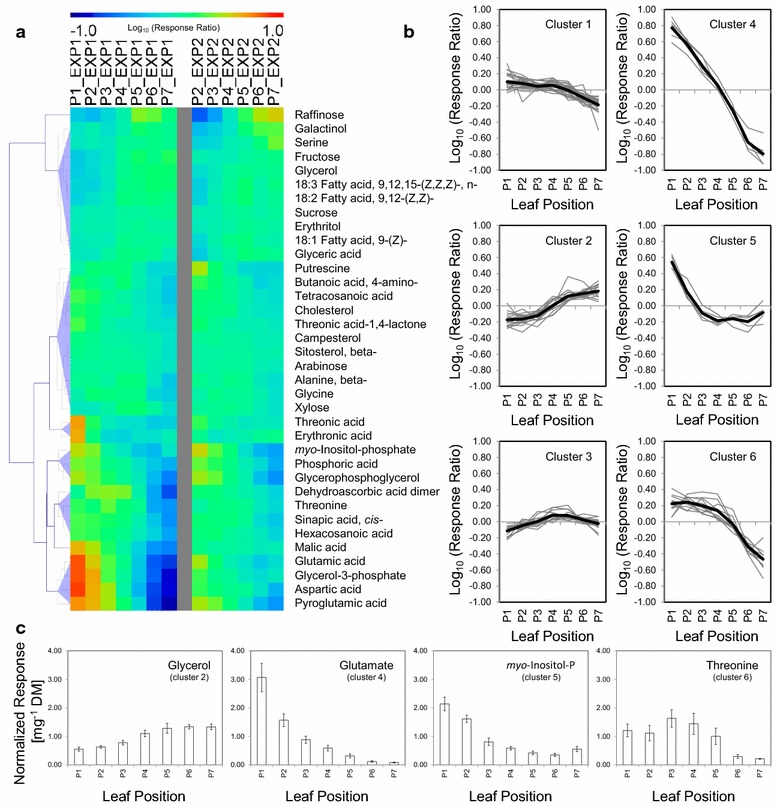
Changes of primary metabolism associated with the sink to source transition of leaves from an *A. thaliana* Col-0 rosette. **a** Heat-map of identified metabolites with robust changes of pool size from leaves of positions P1–P7. Rosettes were at vegetative growth stage 1.12–1.13. Metabolite profiles were normalized to dry mass (DM). Fifty-three of 107 (experiment 1) and 166 (experiment 2) detected metabolites were correlated between the independent experiments (r > 0.6, Pearson’s correlation coefficient) and passed one-way ANOVA and Kruskal–Wallis tests at *P* < 0.05 in at least one experiment (n = 9–10 rosette leaves per position and experiment). The heat-map shows mean centered log_10_-transformed response ratios and hierarchical clustering using Euclidian distance with complete linkage. **b** K-means clusters based on Euclidian distance of mean centered log_10_-transformed response ratios with 7 expected clusters. Cluster 0 that contained constant metabolites is not shown. **c** Normalized responses based on dry mass of 4 representative metabolites from clusters 2, 4, 5, and 6 (mean ± standard error)

Metabolite levels were normalized to dry mass rather than fresh mass. This procedure took into account the general dilution of metabolite pools which was caused by preferential water uptake into leaf tissue at the early leaf expansion stages P1–P3 (Fig. 1b). Primary metabolism was gradually reprogrammed in the course of sink to source transition as was indicated by characteristic metabolic patterns of each leaf position (Fig. 2). The pool sizes of 53 metabolites, 36 of which were identified, changed significantly (*P* < 0.05, Kruskal–Wallis- and ANOVA-tests) with leaf positions (Fig. 2a). The respective patterns of changes were robustly correlated between independent experiments (r > 0.6, Pearson’s correlation coefficient). Thirty-three of the 53 metabolites were highly correlated between independent experiments with r exceeding 0.9 (Additional file 2: Table S2).

These robust metabolic patterns of changes during sink to source transition were grouped into 7 K-means clusters (Fig. 2b). Six clusters represented characteristic metabolite patterns and one cluster contained non-changed metabolites (Additional file 2: Table S2). We annotated multiple metabolites within each cluster (Fig. 2c; Additional file 3: Figure S1). Several patterns of changes were associated with the previously determined transition stage, position P4, specifically in clusters 2, 3, 5, and 6 (Fig. 2c; Additional file 3: Figure S1). The metabolic processes associated with the sink to source transition did not indicate a stepwise transition between 2 states but rather a continuous and gradual metabolic reprogramming. This observation was in agreement with the concept of a gradual transition between young leaves that exhibit predominantly sink characteristics to mature leaves that exhibit predominantly source characteristics [18, 36]. In summary, each leaf position was characterized by a specific metabolic pattern of relative pool size changes (Fig. 2; Additional file 3: Figure S1).

### Development of two ^13^C stable isotope tracing assays for *A. thaliana* rosettes

The conventional metabolomics approach allowed us to identify marker metabolites and metabolic patterns associated with leaves of progressing developmental stages. However, this method did not allow interpretation of the utilization of metabolites. To study how metabolic precursors are utilized in leaves of different developmental stages, we developed ^13^C stable isotope feeding assays to enable primary metabolite profiling of *A. thaliana* plants coupled to simultaneous ^13^C stable isotope tracing.

The feeding assays were developed for the purpose of providing any labeled metabolic precursor molecule to a plant in a way that distributed the labeled precursor rapidly within the whole plant and made it simultaneously available for a wide range of tissues. Several factors have to be taken into account to achieve homogenous feeding. Firstly, the precursor has to be applied by a reservoir which is attached to the plant over the whole time span of an experiment. Thereby, the precursor is constantly available to the plant as a prolonged pulse. Secondly, the concentration of the precursor within the plant will increase until equilibrium with the endogenous unlabeled metabolite is established. In this regard, our assays aim to be similar to leaf disc or conventional petiole feeding assays of dissected leaves. Through these two factors carbon tracing is enabled and the dilution of the label within plant metabolism can be measured by timed snapshots.

**Fig. 3:**
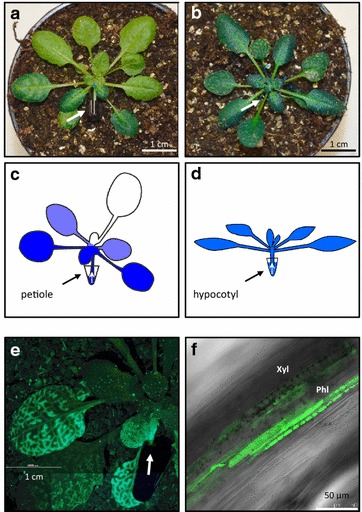
Labeling patterns generated by feeding of dyes through the petiole or the hypocotyl of *A. thaliana* rosettes. Plants were grown on soil under 8 h short day conditions to developmental stage 1.10–1.15. **a** Petiole feeding using a transition leaf between young rosette leaves with sink characteristics and mature source leaves [36]. **b** Hypocotyl feeding using a plant grown under identical conditions. Representative photographs were taken 1 h after start of feeding with a 20 mg mL^−1^ solution of Erioglaucine disodium salt, i.e. Brilliant Blue FCF, in tap water. Arrows indicate the position of the vial that contained the feeding solution. **c** Scheme of the dye-labeling pattern resulting from the petiole feeding assay (PFA). **d** Scheme of the dye-labeling pattern resulting from the hypocotyl-feeding assay (HFA). **e** PFA feeding using 6-Carboxyfluorescein diacetate (CFDA). A section of labeled and non-labeled leaves at different stages is shown. The excitation and emission filters were set to λ = 470 ± 40 nm and λ = 525 ± 50 nm, respectively. The arrow indicates the position of the vial that contained the feeding solution. **f** A longitudinal optical section obtained by confocal laser scanning through a xylem vessel (Xyl) and adjacent CFDA-labeled phloem and phloem companion cells of a petiole (Phl). The excitation and emission filters were set to λ = 488 nm and λ = 560 nm, respectively

To demonstrate our approach, we fed dyes and uniformly labeled [U-^13^C_12_]-sucrose (in the following ^13^C-sucrose) to *A. thaliana* rosettes. Two efficient feeding approaches were explored that both utilize natural transpiration for label distribution: an inverse petiole feeding assay (PFA; Fig. 3a, c) and a hypocotyl feeding assay (HFA; Fig. 3b, d). For the PFA the leaf lamina of one leaf that was attached to a rosette at a specific leaf position was removed and a labeling solution was applied through the remaining petiole that communicated with the rosette and root system. The labeling solution was continuously provided via a 0.1–0.5 mL reservoir. The technical details were similar but miniaturized compared to the feeding of aqueous solutions that was proposed by Lin et al. [39, 40]. A similar feeding strategy of aqueous solutions was applied for the HFA. Here, a complete *A. thaliana* rosette was cut above the root and the labeling solution was applied to the rosette via the hypocotyl. The labeling solution was provided again by a 0.1–0.5 mL reservoir that was placed in the soil below the rosette so as to guarantee instantaneous transfer after the cut. In both assays cutting and transfer were performed in situ, i.e. at the place of the preceding plant cultivation, in order to keep environmental conditions constant and perturbations minimal.

#### Analysis of dye label distribution

To assess the specific features of the two feeding assays, we performed initial test experiments. First we tested homogeneity of the precursor distribution within the rosette. To simulate the specific distribution pattern of each assay we fed a blue dye solution of Brilliant Blue FCF, i.e. Erioglaucine disodium salt. The distribution pattern of PFA showed a gradient across the rosette with strongest staining in the neighboring leaves of the entry petiole. Lowest staining was observed in the leaves opposite to the entry petiole (Fig. 3a, c). This distribution pattern resembled exactly the natural pattern of photosynthetic labeling via a single leaf that was monitored radioactively by ^14^CO_2_ [41]. The blue dye applied to the HFA revealed largely uniform distribution of label across the whole *A. thaliana* rosette (Fig. 3b, d).

To further characterize the transport route of label within the plant rosette, we added a second dye, Carboxyfluorescine diacetate (CFDA). CDFA has been used as a marker of phloem transport [42, 43]. By feeding a mix of Calcofluor White, a marker of the apoplastic continuum [44, 45] that is here used as indicator of xylem transport, and CFDA, we demonstrated that both xylem and phloem were involved in the transport process of labeled molecules within the plant rosette (Additional file 4: Figure S2). Dual transport paths have been reported previously by Lin et al. [39]. Because the label distribution patterns were clearly dependent on vascular connectivity, PFA was suitable only for investigations of 1–2 neighboring leaves from whole plant systems and may include roots or root exudates but not all transition states from sink to source leaves. For analysis of the sink to source continuum we selected the HFA, because of the approximately homogeneous label distribution within the rosette.

#### Analysis of ^13^C label distribution

**Fig. 4:**
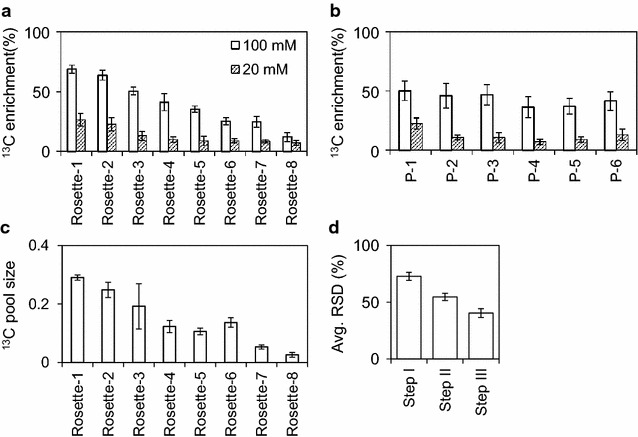
Distribution and reproducibility analysis of labeled sucrose in *A. thaliana* Col-0 rosette applied by the hypocotyl feeding assay. **a** Average ^13^C enrichment of sucrose in rosettes across all leaves (P1–P6). ^13^C enrichment values of single leaves of each rosette were averaged after hypocotyl feeding with a 20 mM and a 100 mM ^13^C-sucrose solution. The range of ^13^C enrichment was 7–26% and 12–68%, respectively. Error bars represent standard deviation (n = 6). **b** Average ^13^C enrichment of sucrose in leave positions across eight rosettes. ^13^C enrichment values of leaf position were averaged after hypocotyl feeding with a 20 mM and a 100 mM ^13^C-sucrose solution. The range of ^13^C enrichment was 7–22% and 36–50%, respectively. Error bars represent standard deviation (n = 8). **c** Average ^13^C pool size (arbitrary units) of sucrose calculated from the sum of labeled and non-labeled sucrose and ^13^C enrichment data. Error bars represent standard error (n = 6). **d** Improvement of averaged relative standard deviation of ^13^C enrichment (avg. RSD %) across 22 annotated and labeled metabolites except sucrose by data processing: (Step I) Relative standard deviation was calculated of each metabolite separately across P1–P3 or P5–P6 leaves and subsequently averaged without further data processing. (Step II) Relative standard deviation of ^13^C enrichment was calculated after signal to noise thresholding obtained by non-labeled control samples. (Step III) Average RSD was further reduced after internal normalization to the ^13^C enrichment value of sucrose in each sample. Error bars represent standard error (n = 22)

In the current study we continued to investigate a series of single leaves (P1–P7) that represented the sink to source transition stages. We chose HFA to achieve approximately homogeneous label distribution and to enable simultaneous sampling of all leaf stages from single rosettes. To further test our experimental setup, we performed HFA with two concentrations of ^13^C-sucrose solutions, 20 and 100 mM, that were applied for 4 h according to the timing of dye labeling. For the quantitative analysis of stable isotope label distribution, we focused on sucrose labeling. First we determined the ^13^C pool size of sucrose, i.e. the part of the metabolite pool size that contains ^13^C. Secondly we calculated the ^13^C enrichment of sucrose, i.e. the percentage of ^13^C in a metabolite pool relative to the sum of labeled and non-labeled carbon. The average ^13^C enrichment of sucrose in rosettes varied after 4 h between individual plants from simultaneously cultivated populations. ^13^C Enrichments ranged from 7 to 26% using 20 mM ^13^C sucrose and from 12 to 68% using a 100 mM solution (Fig. 4a; Additional file 5: Table S3). However, the variance in ^13^C enrichment within single rosettes was low (Fig. 4b; Additional file 5: Table S3). The average ^13^C enrichment of the leaves appeared to be similar across all leaves in agreement with the results obtained by dye feeding (Figs. 3, 4b). We concluded that the feeding variance is mostly plant dependent and only to a small extent dependent on the leaf position. Variation of ^13^C enrichment in the total sucrose pool was mainly caused by differential accumulation of the labeled sucrose precursor rather than by fluctuations of endogenous sucrose levels as was indicated by comparing ^13^C enrichment and ^13^C pool size trends across rosettes (Fig. 4a, c). In conclusion, the plant to plant variation of feeding efficiency in individual rosettes indicated the necessity for eliminating non-labeled outlier samples and a requirement for numerical normalization of sample to sample labeling variance prior to comparative data analysis.

#### Data normalization and signal to noise thresholding

The initial data of annotated metabolites or metabolite fragments after GC–EI–TOF–MS analyses were recorded as nominal mass isotopologue distributions and extracted by routine data processing of all recorded mass features using TagFinder software [46, 47]. The initial mass isotopologue distribution data were corrected for the naturally occurring ^13^C isotope abundance and the contributions of natural isotopes of other elements using the CORRECTOR software package [48]. This software calculates corrected absolute and relative mass isotopologue intensity distributions and ^13^C enrichment values of metabolites or metabolite fragments with defined known molecular formula. For the determination of the total pool size, i.e. the sum of labeled and non-labeled pool size, all detected mass isotopologue intensities of a metabolite or metabolite fragment were summed up. The total pool size values and the ^13^C enrichment percentage output were used to calculate the ^13^C pool size as was detailed above. Besides the corrected errors caused by naturally occurring isotopes, other GC–MS instrument and sample matrix dependent factors can lead to false ^13^C enrichment and ^13^C pool size calculations. In order to find and eliminate such errors, an outlier check was implemented. We introduced a signal to noise threshold of ^13^C enrichment measurements that was based non-labeled control samples. In agreement with previous precision tests of ^13^C-enrichment determinations using GC–MS instruments [6] we applied a 2-times standard error threshold of the value obtained from the non-labeled control group. In most cases this value did not exceed 1%. To further improve data quality, enrichment data of only those metabolite fragments were included that were detected in at least 60% of replicates after signal to noise thresholding. These signal to noise and repeatability checks led to a substantially reduced variance of the data (Fig. 4d). To take the variable tissue delivery of labeled precursor into account and thereby to make the ^13^C data of different rosettes and leaf samples comparable we subsequently normalized all enrichment values obtained from a sample by the ^13^C enrichment of sucrose from the same sample. By this internal standardization of ^13^C enrichment the averaged relative standard deviation of the data was further reduced from 71 to 36% (Fig. 4d; Additional file 5: Table S3).

In summary, the HFA enabled approximately simultaneous delivery of labeled precursor to all leaves of a rosette. Signal to noise thresholding using non-labeled control samples and internal standardization by the measured ^13^C enrichment of the precursor molecule, in our case ^13^C-sucrose, reduced the relative standard deviation that was caused by plant to plant variation of labeling by a factor of two.

### ^13^C Stable isotope tracing reveals differential ^13^C sucrose utilization in sink and source leaves

**Fig. 5:**
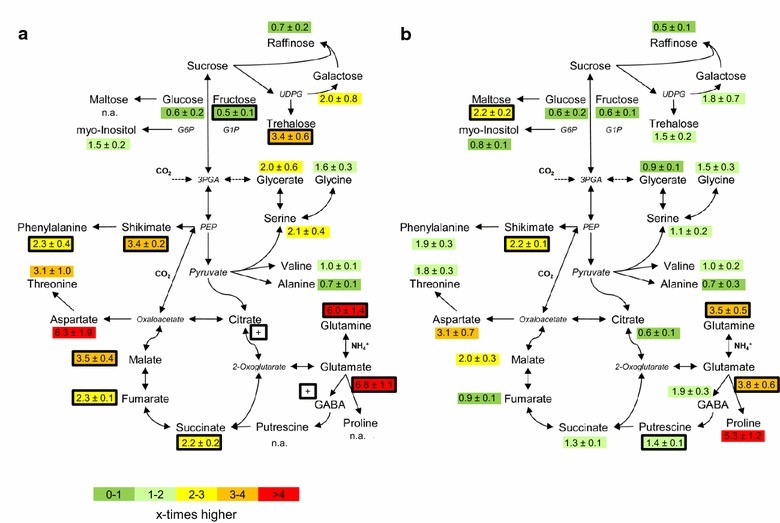
Comparison of the relative changes of metabolites in sink compared to source leaves based on ^13^C labeled (**a)** or total metabolite pool sizes of the same samples (**b**). Complete *A. thaliana* rosettes were labeled 4 h by 100 mM ^13^C sucrose using the HFA. All metabolite pool sizes were normalized to internal standard and sample dry weight [38]. Leaf positions P2–P3 were grouped to represent sink leaves, leaf positions P6–P7 were identified as source leaves. Significance of the sink to source leaf ratios was tested by Student’s *T* test (*P* ≤ 0.01; black boxes). Ratios are means (± standard error), n = 6–10. A metabolite that was labeled only in sink leaves is indicated by (+). Glutamine was determined as sum of glutamine and pyroglutamate

For our test case, we investigated the utilization of uniformly labeled ^13^C sucrose in predominantly sink and predominantly source leaves of the *A. thaliana* rosette. For that purpose, approximately synchronous *A. thaliana* populations were grown to the growth stage 1.12–1.13 as described above. HFA of full rosettes was performed and sampled at 4 h after application of 100 mM ^13^C sucrose solution in tap water. The precursor was applied 2 h after start of the light period. Single leaves of each rosette were harvested from leaf positions P2–P7 and small polar and small lipophilic compounds were measured using GC–EI–TOF–MS metabolite profiling without liquid phase separation as was described previously [38]. The previous growth and metabolite profiling analyses (Figs. 1, 2) and ^13^C enrichment data of a pre-experiment classified the single leaf positions. Hierarchical clustering (Additional file 6: Figure S3) confirmed leaf positions P2–P3 as predominantly sink leaves. These leaves were combined for further analysis (further on referred to as sink leaves). Leaf positions P6–P7 were categorized as predominantly source leaves and also combined for further analysis (further on referred to as source leaves). Our classification of leaf positions according to their growth parameter (Fig. 1), metabolite profiles (Fig. 2), and their ^13^C enrichment patterns after ^13^C sucrose labeling was in agreement with the current concept of a gradual sink to source transition between young and mature leaves (Additional file 6: Figure S3). In the following, we focused on the differential analysis of P2–P3 sink versus P6–P7 source leaves. For clarity the transition state between sink and source at positions P4–P5 was excluded from the current analysis (Additional file 6: Figure S3). In total 23 labeled metabolites were found after data processing steps I-III. Labeled citrate and γ-amino-butyrate (GABA) were only found in sink leaves (Fig. 5a).

First we analyzed which of the metabolite pools received labeled carbon from ^13^C sucrose and if sucrose was differentially utilized in sink compared to source leaves. For this purpose we compared the relative change of total metabolite pool sizes between sink and source leaves (Fig. 5b) to the relative change of the ^13^C pool sizes (Fig. 5a).

An initial comparison of the sink to source ratio of total pool sizes from HFA plants (Fig. 5b) with non-treated plants (Fig. 2; Additional file 2: Table S2) revealed no major differences. Only the sink to source ratio of the total maltose pool was significantly increased in the HFA assay but not in non-treated control plants. Total pool sizes of most metabolites were either increased or unchanged in sink relative to source leaves, except for major carbohydrates such as glucose, fructose, sucrose, or raffinose that had lower total pool sizes in sink leaves (Fig. 5b).

When we compared the sink to source ratios of total pool sizes to the respective ratios of ^13^C pool sizes, we detected characteristic differences. The sink to source ratios based on total and ^13^C pool sizes were similar in many cases, for example, fructose and glucose, the direct cleavage products of sucrose or the amino acids, serine, glycine, valine or alanine (Fig. 5a, b). Three types of deviations were found. First, the sink to source ratio of the total maltose pool increased but maltose did not receive label from ^13^C sucrose. Second, the sink to source ratio of total trehalose did not significantly increase but the ^13^C labeled fraction of trehalose was more than threefold higher in sink compared to source leaves. Third, the total pools of shikimate and phenylalanine as well as total glutamate and glutamine were increased in sink leaves relative to source leaves but the ratios based on the ^13^C pool sizes were even higher. Similarly, except for malate, the total pool sizes of TCA cycle intermediates did not change substantially in sink relative to source leaves, but the ^13^C pool size ratios of succinate, fumarate, and malate were significantly higher and labeled citrate was only detectable in sink leaves but not in source leaves (Fig. 5a).

In conclusion, our simple comparison of the relative changes in sink compared to source leaves that were either based on total or only on the ^13^C labeled pool sizes enabled observations that could not have been made based on the changes of total pool sizes alone. Specifically, the increase of total maltose in sink leaves was not driven by sucrose utilization but by non-labeled endogenous carbohydrate resources. In contrast, ^13^C sucrose was used for trehalose formation preferentially in sink leaves compared to source leaves. Similarly ^13^C sucrose fueled increased production of phenylalanine via shikimate and of glutamate and glutamine in sink leaves. Furthermore, carbon from sucrose was preferentially directed towards TCA cycle intermediates in sink leaves.

### ^13^C stable isotope tracing monitors major paths of carbon through leaf metabolism

Our combined ^13^C tracing and metabolite profiling method was intentionally simplified and does not enable quantification of fluxes. Instead it is intended to detect and quantify relative differences of carbon utilization by following ^13^C dilution along the major paths of carbon from a given metabolic precursor such as ^13^C sucrose. Alternatively to the previous analysis options, ^13^C enrichment of a metabolite pool can be used directly as a numerical distance value between any two metabolites in a metabolic network. Thereby a qualitative interpretation is enabled in regard to the direction of the major carbon flow among all monitored and labeled metabolite pools. For this purpose we chose continuous feeding of labeled sucrose instead of a pulse and chase design. Continuous feeding increases ^13^C enrichment over time until isotopic steady state, i.e. the equilibrium with pre-existing non-labeled carbon sources or de novo assimilation, is reached. We suggest short labeling durations so as to minimize effects that are inevitably caused by the experimental intervention of the assay.

**Fig. 6:**
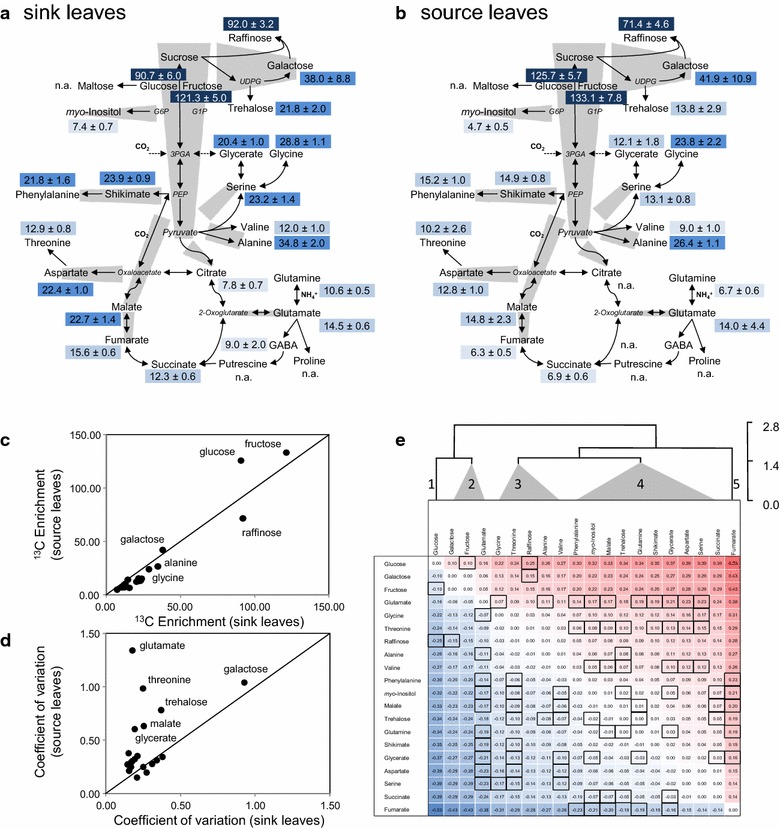
Differential carbon utilization of ^13^C sucrose by sink and source leaf metabolism. Relative ^13^C enrichments of metabolite pools from sink leaves (**a**) or source leaves (**b**) normalized to ^13^C sucrose enrichment per sample (Mean ± standard error, n ≥ 8 mean values per plant of leaves from position P1–P3). Dark to light blue colour coding of relative ^13^C enrichment indicates progressing ^13^C dilution in the analysed metabolite pools (n.a., data not available). Grey trapezoids (isosceles) represent the decrease in ^13^C enrichment between two neighbouring metabolites of the pathway scheme. The length of the trapezoid base is proportional to the ^13^C enrichment of the corresponding metabolite. **c** Bi-plot of relative ^13^C enrichments in sink compared to source leaves. **d** Bi-plot of the coefficients of variation of relative ^13^C enrichment measurements in sink compared to source leaves. **e** Differential matrix of relative ^13^C enrichment in metabolite pools of sink compared to source leaves. For this purpose two distance matrices of ^13^C enrichment ratios among all monitored metabolites were calculated separately from sink and source leaf data (Additional file 9: Table S5). The log_10_-transformed source leaf matrix was subtracted from the log_10_-transformed sink leaf matrix (blue negative, red positive) to obtain the final differential matrix. Significance of the log_10_-transformed ^13^C enrichment ratios within the sink and source leaf matrices was tested by Student’s *t* test (*P* ≤ 0.001, boxes indicate differential ratios that were significant either only in sink or only in source leaves). Hierarchical clustering using Euclidian distance and complete linkage sorted metabolites according to their differential enrichment ratios into five clusters with distance cut-off 1.5. Glutamine was determined as sum of glutamine and pyroglutamate

In our experiments the highest relative ^13^C enrichments compared to sucrose were found in glucose, fructose, galactose and raffinose, in agreement with their short pathway distance from sucrose (Fig. 6a, b; Additional file 7: Table S4). We noticed that direct product metabolites of sucrose, such as fructose, can have ^13^C sucrose corrected, relative ^13^C enrichments even > 100% (Fig. 6a, b). This observation indicated that sucrose and fructose pools were differentially diluted by endogenous non-labeled carbon sources. Likely a metabolically separated non-labeled sucrose pool, e.g., from plant vacuoles, diluted ^13^C sucrose. While the separated non-labeled sucrose pool did not substantially contribute to fructose production, the fed ^13^C sucrose precursor was preferentially used for fructose synthesis. The same observation held true for glucose production in source leaves (Fig. 6b). Next to the main carbohydrates alanine was readily labeled, followed by serine and shikimate in agreement with carbon flow from sucrose through glycolysis and into its amino acid producing side branches. Interestingly, glycine was significantly more ^13^C enriched than serine (Fig. 6a, b). Aspartate and malate had significantly higher ^13^C enrichments than citrate, succinate, or fumarate. This observation indicated direct carbon flow into aspartate and malate from glycolysis through PEP-carboxylase and the oxaloacetate pool that was not detectable by our technology. Considering the increasing ^13^C dilution from malate via fumarate to succinate, the major path of carbon from ^13^C sucrose was apparently inverted in the C4-branch compared to the canonical direction of the TCA cycle. This interpretation was further supported by enhanced abundance of M+3 mass isotopologues within the mass isotopologue distribution patterns (MIDs) of malate and fumarate (Additional file 8: Figure S4). Glutamate and glutamine had higher ^13^C enrichments than citrate. We interpret this finding as a dilution of the citrate ^13^C enrichment measurement, likely by a compartmented non-labeled citrate pool that does not or only very slowly participate in mitochondrial citrate utilization for the production of glutamate and glutamine carbon backbones. This interpretation was supported by the MID of glutamate which had increased M+2 abundance. This observation was consistent with the incorporation of 2 linked carbon atoms from acetyl-CoA into the C6–C4-branch of the TCA cycle (Additional file 8: Figure S4).

### The differential matrix of relative ^13^C enrichment in metabolite pools from sink compared to source leaves reveals differences in carbon partitioning

The comparison of relative ^13^C enrichment of metabolite pools between sink and source leaves showed that carbon utilization from ^13^C sucrose followed the same paths but revealed distinct changes (Fig. 6a, b). As was expected most ^13^C enrichments in source leaves were lower compared to sink leaves (Fig. 6c). In contrast, the coefficient of variation of the ^13^C enrichment measurements in sink leaves was in many cases lower than in source leaves and likely indicative of a tighter control of metabolism in sink leaf tissue (Fig. 6d). The role of sucrose as a carbon and energy source in sink leaves is illustrated by enhanced partitioning of carbon from ^13^C sucrose into amino acid and organic acid pools and reduced partitioning into the major carbohydrates, glucose, fructose and galactose (Fig. 6a, b).

While these global differences of carbon partitioning and respective tissue dependent variation were readily accessible, the details of the changed labeling among all monitored metabolites, for example, the changes of ^13^C enrichment of fumarate or glucose relative to all other metabolites remained hard to judge. For the purpose of more detailed analyses, we developed a differential matrix that compared relative ^13^C enrichment of all monitored metabolites between sink and source leaves (Fig. 6e; Additional file 9: Table S5). We first calculated all ^13^C enrichment ratios across all metabolite pairs separately from the sink leaf and source leaf data, respectively. A log_10_-transformation of these initial matrices generated numerically symmetrical matrices of each tissue type (Additional file 9: Table S5). We subtracted the log_10_-transformed source leaf matrix from the sink leaf matrix and received a differential matrix of relative ^13^C enrichments. This approach demonstrated that glucose had the strongest decrease of carbon partitioning in sink leaves relative to all other metabolites (Fig. 6b, e, cluster 1). In contrast fumarate had the highest relative increase of carbon partitioning (Fig. 6b, e, cluster 5). Hierarchical clustering revealed three additional clusters of metabolites that in sink leaves either received more carbon from ^13^C sucrose in comparison to many other metabolites (Fig. 6b, cluster 4) like fumarate, or received less carbon relative to most metabolites (Fig. 6b, cluster 2 and 3) like glucose.

Through the differential matrix approach further information can be obtained from selected pairs of metabolites that are known to be connected within the metabolic network. For example, glutamate and glutamine are part of the glutamine synthase (GS)/glutamine-2oxoglutarate-aminotransferase (GOGAT) cycle of N-assimilation. In sink leaves more carbon is partitioned into the glutamine pool relative to glutamate (Fig. 6e, row “glutamate”, glutamine value: + 0.19). Based on this observation the GS/GOGAT cycle appeared to be activated towards glutamine synthesis in sink leaves. As a second example, threonine as part of the aspartate amino acid biosynthesis family is synthesized through several steps from aspartate. In sink leaves less carbon is partitioned into threonine relative to aspartate (Fig. 6e, row “aspartate”, threonine value: − 0.14). Thus threonine synthesis from aspartate in sink leaves appeared to be reduced in comparison to source leaves.

In conclusion, we demonstrated that hypotheses on the activation of reactions or reaction sequences in an investigated tissue (sink leaves) compared to a reference tissue (source leaves) can be obtained. But we also caution. The ^13^C enrichment values and ratios derived from ^13^C enrichment data lack the information of absolute metabolite concentrations. Therefore ^13^C enrichment data can only be used to estimate the relative changes of carbon partitioning from a precursor into a product metabolite, but in vivo reaction rates (fluxes) cannot be calculated without additional quantification of the pool sizes. In addition, the interpretation of altered carbon partitioning in a complex system like the *A. thaliana* leaf requires consideration of ^13^C dilution effects by non-labeled metabolite pools that result from subcellular compartmentation that we did not resolve in this study. In the following we will highlight and integrate selected findings of our study.

## Discussion

### The tricarboxylic acid (TCA) cycle

Conventional metabolomics studies tend to associate relative changes of the pools of TCA cycle intermediates with deductions of altered carbon flux through the cycle for the purpose of providing the carbon back bones for amino acid biosynthesis and possibly for the support of altered energy demands of an investigated tissue. In our test system, namely the sink to source transition during *A. thaliana* leaf development we indeed expected enhanced partitioning of carbon from sucrose towards TCA cycle intermediates in the actively growing sink tissue (Fig. 1), but except for malate the analysis of the relative changes of total citrate, succinate or fumarate pool sizes did not reflect these expectations (Fig. 5b). However, already the analysis of the relative changes of the sucrose-derived ^13^C pool sizes clearly revealed enhanced partitioning of carbon towards malate, fumarate, and succinate in sink leaves and also citrate (Fig. 5a). The comparison of relative ^13^C enrichment in the respective pools clearly supported the interpretation of enhanced carbon partitioning into these pools (Fig. 6a, b) and in addition demonstrated increased use of carbon for aspartate, glutamate and glutamine production. Finally the differential matrix of relative ^13^C enrichment revealed that succinate and especially fumarate had the highest increases of carbon partitioning in sink leaves relative to all other measured metabolites (Fig. 6e, bottom rows).

The comparison of relative ^13^C enrichment (Fig. 6a, b) indicated in addition that the TCA cycle may operate in a non-cyclic flux mode as has been postulated for illuminated leaves [49–51]. In this mode the C4 TCA-cycle branch, i.e. oxaloacetate, malate, fumarate, and succinate, may be only weakly connected to the decarboxylating C6–C4 branch, i.e. citrate, aconitate, isocitrate, 2-oxoglutarate, succinyl-CoA. Our data support the non-cyclic flux mode hypothesis for the C4 branch, because relative ^13^C enrichment analysis in source and especially in sink leaves indicated a predominant carbon flux from glycolysis to malate and aspartate followed by fumarate and succinate (Fig. 6a, b). We, however, caution that this interpretation of our data is only valid at whole tissue level and cannot be applied without additional analyses to the relatively small compartmented mitochondrial TCA cycle pools. A likely cause of our observations is an activated flux through the anaplerotic phosphoenolpyruvate carboxylase reaction in sink leaves that extends towards fumarate and succinate [50]. The C6–C4 branch may be fueled mainly by non-labeled, stored, cytosolic, or vacuolar citrate [27, 51]. In sink leaves de novo synthesis of citrate is apparently activated (Fig. 6a). The presence of large citrate stores and activation of citrate synthesis in sink leaves may be explained by the role of cytosolic ATP citrate lyase (ACL) [24, 52] and other ACLs that are regulated by sink strength [53] for the balanced synthesis of acetyl CoA in all subcellular compartments during plant development [54]. The observation that sucrose-derived carbon in sink leaves is preferentially partitioned into leaf fumarate was a surprising observation, especially in view of the constant total pool size (Fig. 5b). Activated fumarate and also succinate or malate synthesis may be explained by a function of these acids as additional intermediate carbon stores that may maintain leaf growth throughout the diurnal cycle [55]. Fumarate can be transported [56] and may accumulate to a large extent. Under starch depletion conditions, for example at the end of a long night, fumarate can serve as a substrate for respiration [55].

### Nitrogen assimilation and amino acid biosynthesis

Within an actively growing tissue such as sink leaves we expect activation of amino acid biosynthesis for protein biosynthesis [36]. At the level of changes of total pool sizes we observed significant (*P* < 0.01) > threefold increases of glutamine and glutamate (Fig. 5b). Likewise aspartate was > threefold increased but only at *P* = 0.06 (Additional file 7: Table S4). In agreement with an increased demand for nitrogen assimilation in sink tissue the respective changes of the ^13^C pool sizes were approximately doubled (Fig. 5b). Both in sink and source tissue the carbon back bones of glutamine and glutamate are derived also from de novo synthesized citrate (Fig. 6a, b). Whereas the carbon partitioning into aspartate is clearly enhanced in sink leaves (Fig. 6a), the partitioning into the GS/GOGAT cycle remained approximately constant, albeit with a preference towards glutamine. We interpret the approximately constant carbon partitioning into the GS/GOGAT cycle by equal increases of the utilization of de novo synthesized and stored citrate for glutamine and glutamate carbon back bone synthesis in sink leaves.

The total pool size changes of other amino acids remained approximately constant, indicative of biosynthesis of amino acids that are immediately consumed for protein production (Fig. 5b). Similarly the ^13^C pool size changes did not largely deviate from the total pool size changes (Fig. 5a). In agreement with activated amino acid biosynthesis the relative ^13^C enrichment of essentially all measured amino acids increased (Fig. 6a–c). Specifically the shikimate pathway of aromatic amino acid biosynthesis was activated, an observation that may be explained by additional demand for phenylpropanoids [57], for example, for the purpose of lignin biosynthesis that is required for the formation of vasculature and secondary cell walls during leaf growth [58, 59]. An additional surprising observation was the fact that in both sink and source leaves glycine had significantly higher relative ^13^C enrichment compared to serine or glycerate (Fig. 6a, b). This finding indicated that glycine was not preferentially synthesized from serine but rather that the glycine pool received sucrose-derived carbon preferentially through other pathways. We can currently only speculate that either glycine may be synthesized via a compartmented path via threonine and threonine aldolase [60] or alternatively that glycine may receive label from sucrose-derived glycolate or glyoxylate. Photorespiratory origin of labeled glycine appears to be unlikely since CO_2_ assimilation by ribulose-1,5-bisphosphate carboxylase oxygenase that in our assays competes with the ^13^C sucrose feeding should preferentially produce non-labeled glycine.

## Conclusions

We developed rapid ≤ 4 h stable isotope feeding assays for small labeled metabolic precursors that allow the paralleled monitoring of the relative changes of metabolite pool sizes in combination with the metabolic utilization of the labeled atoms from the fed precursor metabolite. The test case analysis of the classical sink to source transition paradigm of leaf development using the *A. thaliana* rosette model clearly indicated that the combined method enables insights that are beyond conventional metabolite profiling experiments and allow conclusions and discoveries to be made in regard to differential carbon partitioning that remain obscured or hidden in conventional metabolomics studies. Our study indicated that whole tissue analysis of organic acids such as citrate, fumarate, or malate monitored not the relatively small TCA cycle pools but rather larger extra-mitochondrial organic acid buffers that can be generated from carbohydrates by cytosolic or plastid PEPC reactions and used for TCA cycle anaplerosis by non-cyclic flux modes. For example, stored citrate may be used to fuel the GS/GOGAT cycle in growing tissue or fumarate to drive respiration under conditions of temporary carbohydrate shortage [61, 62].

Thus we present a rapid screening method for altered metabolic fluxes that fills the gap to but does not replace demanding and time consuming flux measurements and modeling approaches. In view of the current and future sensitivity increases of high mass resolution metabolomics methods, e.g., [63, 64], and the development of algorithms to detect unknown labeled metabolites [65], we are convinced of the high potential of our method for the rapid screening of carbon partitioning that can be easily varied and extended by feeding other carbon or nitrogen labeled metabolic precursors.

## Methods

### Plant growth, leaf staging, and growth analysis

Seed batches of *A. thaliana* Col-0 were stored dry at 4 °C for at least 1 month prior to the experiments performed in this study. Seeds were sterilized for 3–15 min at room temperature with a 70% (v/v) ethanol solution containing 0.5% (w/v) Triton X-100. Seeds were germinated after a 95% ethanol rinse and complete evaporation of residual ethanol. Germination was without further stratification on MS agar plates which were supplemented with 0.8% (w/v) agar and 2% (w/v) sucrose [66]. Germinating seeds were kept for 8 days at constant 22 °C temperature under a 16 h/8 h day/night cycle with 120 µmol m^−2^ s^−1^ photosynthetic photon flux (PPF) during the day. Seedlings were transferred to soil at stage ~ 1.02 [34] and cultivated in plastic pots of 6–10 cm diameter which were filled with Standard Kompost Erde (Stender AG, Schermbeck, Germany). Subsequently the plants were grown in a growth chamber for 4 weeks under an 8 h/16 h day/night cycle with 120 µmol m^−2^ s^−1^ PPF during the day. Day temperature was set to 20 °C. The night temperature was 18 °C. During the first of the 4 weeks seedlings were kept under a transparent plastic hood to achieve a gradual acclimation from high humidity during in vitro germination to lower air humidity and higher air movement in the growth chamber.

Uniform anatomical, developmental, and physiological leaf stages were obtained by a staging system that started with position P1, i.e. the chronologically youngest fully visible leaf in the center of the rosette. Successive older leaves, leaf positions P2 to P9, are shifted by ~ 137.5° angles, respectively (Fig. 1a). Most rosettes had a counter-clockwise circular pattern. A minority of individuals which was not further analyzed in this study followed a clockwise pattern. Positions > P9 and cotyledons were not analyzed.

The ratio of dry mass to fresh mass was determined gravimetrically. Three independent pools of ~ 20 leaves from each position were harvested and weighed immediately and after reaching constant weight in a compartment dryer. Leaf length and fresh mass at each position were determined by paired measurements of full sets of P1–P9 leaves from 68 plants. Morphometric analyses of leaf length were performed as was described earlier [67]. Digital photographs were taken at 4288 × 2848 pixel resolution using a SLR Nikon D5000 camera (Nikon Cooperation, Tokyo, Japan), a AF-S DX Nikkor 18–55 mm, F/3.5–5.6 VR lens and a 1.0 mm resolution scale. The length of a leaf from the center of a rosette to leaf tip was determined manually supervised using ImageJ software version 1.46r, http://rsbweb.nih.gov/ij/index.html [68]. The dry mass of single leaves was calculated based on leaf length measurements using position-specific dry to fresh mass ratios (Fig. 1b) and position specific linear regression functions. The regression functions for this projection were obtained after outlier removal from 66 to 68 paired measurements with r^2^ = 0.813 ± 0.036. The rate of longitudinal growth and projected accumulation of dry mass per day was determined over an interval of 3 days prior to the day of harvest. The relative gain of leaf length and dry mass per day was expressed as percentage relative to the length and mass at the day of harvest.

### Statistical analysis and visualization of numerical data

Box-plot analyses were generated using the R-Studio software, version 0.97.336 [69], using the software and algorithm package, R-2.15.2 [70]. Multi-Experiment Viewer Software was used for hierarchical clustering analysis, ANOVA and Kruskal–Wallis test [71] [MeV v4.7.4 Multi-Experiment Viewer; (http://www.tm4.org/mev)]. All other statistical analyses were performed with Microsoft Excel 2010 (Microsoft Excel, Redmond, Washington, USA).

### Profiling of metabolite pool sizes

A fraction enriched for primary metabolites was profiled after chemical derivatization, i.e. methoxyamination and trimethysilylation, using GC–MS technology [1, 72]. Throughout this study exclusively paired leaves of single plant rosettes were harvested and analyzed. Leaves were not pooled prior to analysis. P1–P7 or P1–P9 leaves were harvested from each rosette and metabolically inactivated by shock freezing in liquid nitrogen. Fresh weight (FW) was determined gravimetrically in the frozen state after harvesting. The minimum weight that was accurately determined, i.e. relative standard deviation < 10%, while frozen was 2.5 mg [38]. Several modifications were introduced to optimize the comparative profiling of single leaves in the fresh mass range 2.5–50.0 mg [38].

Each leaf was ground and extracted with 400 µl of a methanol mix [38]. The extraction volume was adapted to the measured FW, using 400 µl for a FW range of 2.5–10 mg, and 800 µl for 10–20 mg, 1200 µl for 20–30 mg, and proportional up-scaling in 10 mg steps. For the extraction a methanol mix was prepared with 398 µl methanol, 1 µl of a 0.2 mg mL^−1^ uniformly labeled U-^13^C_6_-sorbitol (in the following ^13^C-sorbitol) solution and 1 µl of a 2 mg mL^−1^ (CHCl_3_) nonadecanoic acid methyl ester as internal standards. Samples were extracted for 15 min at 70 °C in a ThermoShaker. Aliquots of 350 µl were obtained after centrifugation, avoiding contamination by cellular debris. The aliquots were dried by vacuum concentration and stored dry under inert gas at − 20 °C until further processing.

Metabolite profiling was performed as detailed previously [73, 74] by gas chromatography coupled to electron impact ionization/time-of-flight mass spectrometry (further GC–MS) using an Agilent 6890N24 gas chromatograph (Agilent Technologies, Böblingen, Germany; http://www.agilent.com) with split and splitless injection onto a FactorFour VF-5ms capillary column, 30 m length, 0.25 mm inner diameter, 0.25 μm film thickness (Varian-Agilent Technologies), which was connected to a Pegasus III time-of-flight mass spectrometer (LECO Instrumente GmbH, Mönchengladbach, Germany; http://www.leco.de).

Dried metabolites were methoxyaminated and trimethylsilylated manually prior to GC–MS analysis [1, 72–75]. Retention indices were calibrated by addition of a C_10_, C_12_, C_15_, C_18_, C_19_, C_22_, C_28_, C_32_, and C_36_ *n*-alkane mixture to each sample [76].

GC–MS chromatograms were acquired, visually controlled, baseline-corrected, and exported in NetCDF file format using ChromaTOF software (Version 4.22; LECO, St. Joseph, USA). GC–MS data processing into a standardized numerical data matrix and compound identification were performed using the TagFinder software [46, 47]. Compounds were identified by mass spectral and retention time index matching to the reference collection of the Golm Metabolome Database (GMD, http://gmd.mpimpgolm.mpg.de/) [77–79] and the mass spectra of the NIST08 database (http://www.nist.gov/srd/mslist.htm). Guidelines for manually supervised metabolite identification were the presence of at least 3 specific mass fragments per compound and a retention index deviation < 1.0% [76].

The measured raw intensities of all mass features of an experiment were normalized by sample dry weight, internal standard and maximum scaled. For quantification purposes all mass features were evaluated for the best specific, selective and quantitative representation of observed analytes. Laboratory and reagent contaminations were evaluated by non-sample control experiments. Metabolites were routinely assessed by relative changes expressed as response ratios, i.e. x-fold factors in comparison to the overall median of each metabolite measure.

### *A. thaliana* feeding assays and sampling

Feeding assays were performed using dissected bottoms of black 0.5 mL Eppendorf tubes that were cut off to 4–5 mm size and used as disposable reservoirs for labeling solution. Labeling solution was prepared as stock solutions, 100 mM or 20 mM ^13^C sucrose in tap water. Do not use distilled or deionized water. A defined volume of labeling solution was filled into the reservoir for each assay (30 µL for PFA; 100 µL for HFA).

#### Petiole feeding assay (PFA)

The leaf lamina of the “feeding” leaf was removed by a clear scalpel cut and the reservoir and labeling solution immediately placed to cover the petiole stump of the remaining petiole that was still attached to the plant. The solution covered the cut completely and entered the plant via the petiole. The solution stayed even in tilted reservoirs because of the capillary forces of small aqueous volumes. The solution was provided in excess of the transpired volume for the experiment duration. Care was taken to avoid exposure of the cut to air both at the start and the end of the labeling time.

#### Hypocotyl feeding assay (HFA)

The reservoir was placed vertically in the soil close to the remaining root. Care was taken that soil did not contaminate the labeling solution and that no soil fibers drained the reservoirs by capillary forces in the course of the experiment. The hypocotyl of fed plants was cut immediately above the root and placed instantaneously into the filled reservoir. Cutting and placing the plants into the solution should not exceed 1–2 s. Care was taken to avoid even slightly prolonged exposure of the cut to air. Plants that showed indications of wilting after labeling were excluded.

For both assays plants were incubated for 4 h in the labeling solution. During the preparation and the whole assay plants stayed in the climate chamber with unchanged environmental conditions. At the end of an experiment leaves were harvested successively from leaf position P1 to P9, directly placed into round bottom 2 mL Eppendorf tubes and immediately frozen in liquid nitrogen to stop metabolism. Leaves were weighed in the frozen state and stored in − 80 °C freezer until further processing.

### Processing and evaluation of ^13^C labeling data

Labeling data were extracted from the GC–MS recordings after baseline correction of raw intensities from mass features that represented annotated analyte fragments and their nominal mass isotopologues. Typically at least three mass fragments of each analyte and their isotopologue distributions were processed if available. To correct isotopologue intensities for the contribution of naturally occurring isotopes [80, 81] data were further processed with the CORRECTOR software tool [48, 82]. The CORRECTOR calculates ^13^C enrichments, corrected isotopologue intensities. The CORRECTOR version (v1.91) was used (http://www.mpimp-golm.mpg.de/10871/Supplementary_Materials).

From the corrected data the “total pool size” value of each metabolite was calculated by summation of all measured isotopologue intensities. If multiple analyte fragments of single metabolites were available each analyte fragment and respective isotopologue distribution was processed separately and normalized by dry weight (DW) and internal standard ^13^C-sorbitol. Endogenous sorbitol was below detection limit in the analyzed samples. To test the quality of the selected metabolite fragments the total pool size measures of all available metabolite fragments were correlated across all samples. Fragments that did not correlate, i.e. Pearson’s correlation coefficient ≤ 0.900, were removed from further analysis.

The initial ^13^C enrichment values, data processing step I (Fig. 4d; Additional file 7: Table S4), were quality tested and signal to noise filtered. To determine the lower limit of an acceptable positive ^13^C enrichment value, a threshold ^13^C enrichment value was measured using non-labeled control samples that were included into the experimental design of each labeling study. The threshold was defined as average ^13^C enrichment of non-labeled control samples plus twofold of the determined standard error. Values below threshold were removed from further analysis. In addition, a coverage > 60% of all replicate samples was required for further processing, data processing step II (Fig. 4d; Additional file 7: Table S4). If multiple fragments that represented the same metabolite remained, only one with best coverage of replicates samples was further processed. The signal to noise and replication filtered ^13^C-enrichment data were subsequently normalized to the ^13^C-enrichment of sucrose within each sample so as to generate internally standardized “relative ^13^C enrichment” values, data processing step III (Fig. 4d; Additional file 7: Table S4). Note that absolute ^13^C-enrichment data cannot exceed 100%, but “relative ^13^C enrichment” after internal normalization by ^13^C-enrichment of sucrose may be > 100%. The “^13^C pool size” value of metabolites was defined as the product of “total pool size” and “relative ^13^C enrichment” (Additional file 7: Table S4).

## Additional files

**Additional file 1: Table S1.** Growth and developmental data that characterize the sink to source transition of rosette leaves from whole plant stage 1.12–1.13 cultivated under an 8 h short day regime.

**Additional file 2: Table S2.** Metabolic data that characterize the sink to source transition of rosette leaves from whole plant stage 1.12–1.13 cultivated under an 8 h short day regime.

**Additional file 3: Figure S1.** Examples of metabolites with pool size changes that are associated with the sink to source transition of leaves from an *A. thaliana* Col-0 rosette at vegetative growth stage 1.12–1.13. Normalized responses based on dry mass (mean ± standard error, n = 9–10). The mass spectra, retention indices, and updated annotations can be accessed through the Golm Metabolome Database (http://gmd.mpimp-golm.mpg.de/).

**Additional file 4: Figure S2.** Simultaneous labeling of vascular tissue by co-feeding of 6-Carboxyfluorescein diacetate (green fluorescence) and Calcofluor White (blue fluorescence) dissolved in tap water. The fluorescent dyes were fed through the petiole of a transition leaf. *A. thaliana* plants were grown on soil under 8 h short day conditions and analysed at developmental stage 1.10–1.15. (A) Bright-field image of an approximately longitudinal optical section obtained by a confocal laser scanning microscope. The arrow indicates the position of a xylem vessel. (B) Phloem tissue indicated by 6-Carboxyfluorescein flourescence using excitation wave length λ = 488 nm and emission filter λ = 560 nm (green). (C) Xylem and apoplastic continuum indicated by Calcofluor White fluorescence using excitation wave length λ = 355 nm and an emission filter λ = 425 nm (blue). (D) Merged images (A–C).

**Additional file 5: Table S3.** Numerical data of Fig. 4.

**Additional file 6: Figure S3.** Hierarchical clustering of *A. thaliana* leaf positions P2–P7 according to the ^13^C-labeling of metabolites 4 h after application of ^13^C sucrose using the HFA. Only metabolites that were labeled at all leaf positions were clustered using the Pearson’s correlation distance metric and complete linkage. Note that the labeling patterns of P2–P3 and P6–P7 were highly similar in contrast to the transition stage P4–P5. *Glutamine was determined as sum of glutamine and pyroglutamate

**Additional file 7: Table S4.** Numerical data of Fig. 6a–d.

**Additional file 8: Figure S4.** Qualitative analysis of mass isotopologue distributions (MIDs) of metabolites from the TCA cycle in sink leaves. Graphs show MIDs after correction for naturally occurring isotopes. Fragments were selected to represent MIDs of the complete carbon backbone as indicated below MIDs. The full MIDs of such fragments of aspartate and alanine were not detectable due to low fragment abundance. Instead we choose for comparison abundant fragments of aspartate and alanine that contained only part of the carbon backbone. For qualitative analysis we choose samples with typical low (blue) or medium (red) ^13^C enrichment that is indicated by inserts. Note that glutamate shows an increased abundance of M+2 (black arrow) that indicates preferred incorporation of 2 linked ^13^C atoms via acetyl-CoA. Malate and fumarate have enhanced M+3 (black arrows) relative to almost non-detectable M+4 that are consistent with preferred ^12^CO_2_ incorporation via the PEP carboxylase reaction.

**Additional file 9: Table S5.** Numerical data and calculations of Fig. 6e.

## Abbreviations

PFA: petiole feeding assay
HFA: hypocotyl feeding assay
GC: gas chromatography
MS: mass spectrometry
LC: liquid chromatography
ANOVA: analysis of variance
GMD: Golm Metabolome Database

## References

[1] Fiehn O, Kopka J, Dörmann P, Altmann T, Trethewey RN, Willmitzer L (2000). Metabolite profiling for plant functional genomics. Nat Biotechnol.

[2] Kaplan F, Kopka J, Haskell DW (2004). Exploring the temperature-stress metabolome of Arabidopsis. Plant Physiol.

[3] Klotke J, Kopka J, Gatzke N, Heyer AG (2004). Impact of soluble sugar concentrations on the acquisition of freezing tolerance in accessions of Arabidopsis thaliana with contrasting cold adaptation—evidence for a role of raffinose in cold acclimation. Plant Cell Environ.

[4] Stitt M, Baker NR (1996). Metabolic regulation of photosynthesis. Photosynthesis and the environment.

[5] Williams TCR, Miguet L, Masakapalli SK, Kruger NJ, Sweetlove LJ, Ratcliffe RG (2008). Metabolic network fluxes in heterotrophic Arabidopsis cells: stability of the flux distribution under different oxygenation conditions. Plant Physiol.

[6] Huege J, Sulpice R, Gibon Y, Lisec J, Koehl K, Kopka J (2007). GC-EI-TOF-MS analysis of in vivo carbon-partitioning into soluble metabolite pools of higher plants by monitoring isotope dilution after ^13^CO_2_ labelling. Phytochemistry.

[7] Calvin M. The photosynthetic carbon cycle. J Chem Soc. 1956;1895-915.

[8] Schaefer J, Kier LD, Stejskal EO (1980). Characterization of photorespiration in intact leaves using (13)carbon dioxide labeling. Plant Physiol.

[9] Vernon LP, Aronoff S (1952). Metabolism of soybean leaves. IV. Translocation from soybean leaves. Arch Biochem Biophys.

[10] Giersch C, Heber U, Kaiser G, Walker DA, Robinson SP (1980). Intracellular metabolite gradients and flow of carbon during photosynthesis of leaf protoplasts. Arch Biochem Biophys.

[11] Kaiser G, Martinoia E, Wiemken A (1982). Rapid appearance of photosynthetic products in the vacuoles isolated from barley mesophyll protoplasts by a new fast method. Z für Pflanzenphysiol.

[12] Kaiser G, Heber U (1984). Sucrose transport into vacuoles isolated from barley mesophyll protoplasts. Planta.

[13] Ludewig F, Flügge UI (2013). Role of metabolite transporters in source-sink carbon allocation. Front Plant Sci.

[14] Aronoff S (1955). Translocation from soybean leaves. II. Plant Physiol.

[15] Turgeon R, Webb JA (1973). Leaf development and phloem transport in *Cucurbita pepo*: Transition from import to export. Planta.

[16] Lemoine R, Camera SL, Atanassova R, Dédaldéchamp F, Allario T, Pourtau N (2013). Source-to-sink transport of sugar and regulation by environmental factors. Front Plant Sci.

[17] Fellows RJ, Geiger DR (1974). Structural and physiological changes in sugar beet leaves during sink to source conversion. Plant Physiol.

[18] Turgeon R (1989). The sink-source transition in leaves. Annu Rev Plant Physiol Plant Mol Biol.

[19] Fernie AR, Geigenberger P, Stitt M (2005). Flux an important, but neglected, component of functional genomics. Curr Opin Plant Biol.

[20] Baxter CJ, Redestig H, Schauer N, Repsilber D, Patil KR, Nielsen J (2007). The metabolic response of heterotrophic Arabidopsis cells to oxidative stress. Plant Physiol.

[21] Allen DK, Libourel IGL, Shachar-Hill Y (2009). Metabolic flux analysis in plants: coping with complexity. Plant Cell Environ.

[22] Roscher A, Kruger NJ, Ratcliffe RG (2000). Strategies for metabolic flux analysis in plants using isotope labelling. J Biotechnol.

[23] Nargund S, Sriram G (2013). Designer labels for plant metabolism: statistical design of isotope labeling experiments for improved quantification of flux in complex plant metabolic networks. Mol Biosyst.

[24] Junker BH, Lonien J, Heady LE, Rogers A, Schwender J (2007). Parallel determination of enzyme activities and in vivo fluxes in *Brassica napus* embryos grown on organic or inorganic nitrogen source. Phytochemistry.

[25] Roessner-Tunali U, Liu J, Leisse A, Balbo I, Perez-Melis A, Willmitzer L (2004). Kinetics of labelling of organic and amino acids in potato tubers by gas chromatography-mass spectrometry following incubation in ^13^C labelled isotopes. Plant J.

[26] Masakapalli SK, Le Lay P, Huddleston JE, Pollock NL, Kruger NJ, Ratcliffe RG (2010). Subcellular flux analysis of central metabolism in a heterotrophic Arabidopsis cell suspension using steady-state stable isotope labeling. Plant Physiol.

[27] Szecowka M, Heise R, Tohge T, Nunes-Nesi A, Vosloh D, Huege J (2013). Metabolic fluxes in an illuminated Arabidopsis rosette. Plant Cell.

[28] Wichern F, Mayer J, Joergensen R, Müller T (2010). Evaluation of the wick method for in situ ^13^C and ^15^N labelling of annual plants using sugar-urea mixtures. Plant Soil.

[29] Morris DA, Kadir G (1972). Pathways of auxin transport in the intact pea seedling (*Pisum sativum* L.). Planta.

[30] Jamet E, Kopp M, Fritig B (1985). The pathogenesis-related proteins of tobacco—their labeling from ^14^C amino acids in leaves reacting hypersensitively to infection by tobacco mosaic-virus. Physiol Plant Pathol.

[31] Timm S, Nunes-Nesi A, Pamik T, Morgenthal K, Wienkoop S, Keerberg O (2008). A cytosolic pathway for the conversion of hydroxypyruvate to glycerate during photorespiration in Arabidopsis. Plant Cell..

[32] Zook M, Hammerschmidt R (1997). Origin of the thiazole ring of camalexin, a phytoalexin from *Arabidopsis thaliana*. Plant Physiol.

[33] Geigenberger P, Stitt M (1991). A, “futile” cycle of sucrose synthesis and degradation is involved in regulating partitioning between sucrose, starch and respiration in cotyledons of germinating *Ricinus communis* L. seedlings when phloem transport is inhibited. Planta.

[34] Boyes DC, Zayed AM, Ascenzi R, McCaskill AJ, Hoffman NE, Davis KR (2001). Growth stage–based phenotypic analysis of Arabidopsis: A model for high throughput functional genomics in plants. Plant Cell.

[35] Berardini TZ, Bollman K, Sun H, Poethig RS (2001). Regulation of vegetative phase change in *Arabidopsis thaliana* by cyclophilin 40. Science.

[36] Pantin F, Simonneau T, Muller B (2012). Coming of leaf age: Control of growth by hydraulics and metabolics during leaf ontogeny. New Phytol.

[37] Watanabe M, Balazadeh S, Tohge T, Erban A, Giavalisco P, Kopka J (2013). Comprehensive dissection of spatiotemporal metabolic shifts in primary, secondary, and lipid metabolism during developmental senescence in Arabidopsis. Plant Physiol.

[38] Dethloff F, Erban A, Orf I, Alpers J, Fehrle I, Beine-Golovchuk O (2014). Profiling methods to identify cold-regulated primary metabolites using gas chromatography coupled to mass spectrometry. Methods Mol Biol.

[39] Lin YH, Ferguson BJ, Kereszt A, Gresshoff PM (2010). Suppression of hypernodulation in soybean by a leaf-extracted, NARK- and Nod factor-dependent, low molecular mass fraction. New Phytol.

[40] Lin Y-H, Lin M-H, Gresshoff PM, Ferguson BJ (2011). An efficient petiole-feeding bioassay for introducing aqueous solutions into dicotyledonous plants. Nat Protoc.

[41] Kolling K, Muller A, Flutsch P, Zeeman SC (2013). A device for single leaf labelling with CO_2_ isotopes to study carbon allocation and partitioning in *Arabidopsis thaliana*. Plant Methods.

[42] Oparka CMD, Prior OAM, Fisher DB (1994). Real-time imaging of phloem unloading in the root tip of Arabidopsis. Plant J.

[43] Wright KM, Oparka KJ (1997). Metabolic inhibitors induce symplastic movement of solutes from the transport phloem of Arabidopsis roots. J Exp Bot.

[44] Coetzee J, Fineran BA (1987). The apoplastic continuum, nutrient absorption and plasmatubules in the dwarf mistletoe *Korthalsella lindsayi* (Viscaceae). Protoplasma.

[45] Shatil-Cohen A, Moshelion M (2012). Smart pipes. Plant Signal Behav.

[46] Lüdemann A, Strassburg K, Erban A, Kopka J (2008). TagFinder for the quantitative analysis of gas chromatography-mass spectrometry (GC-MS)-based metabolite profiling experiments. Bioinformatics.

[47] Allwood JW, Erban A, de Koning S, Dunn WB, Luedemann A, Lommen A (2009). Inter-laboratory reproducibility of fast gas chromatography-electron impact-time of flight mass spectrometry (GC-EI-TOF/MS) based plant metabolomics. Metabolomics.

[48] Huege J, Goetze J, Dethloff F, Junker B, Kopka J (2014). Quantification of stable isotope label in metabolites via mass spectrometry. Methods Mol Biol.

[49] Sweetlove LJ, Beard KFM, Nunes-Nesi A, Fernie AR, Ratcliffe RG (2010). Not just a circle: Flux modes in the plant TCA cycle. Trends Plant Sci.

[50] Tcherkez G, Mahé A, Gauthier P, Mauve C, Gout E, Bligny R (2009). In folio respiratory fluxomics revealed by ^13^C isotopic labeling and H/D isotope effects highlight the noncyclic nature of the tricarboxylic acid “cycle” in illuminated leaves. Plant Physiol.

[51] Gauthier PPG, Bligny R, Gout E, Mahé A, Nogués S, Hodges M (2010). In folio isotopic tracing demonstrates that nitrogen assimilation into glutamate is mostly independent from current CO_2_ assimilation in illuminated leaves of *Brassica napus*. New Phytol.

[52] Schwender J, Shachar-Hill Y, Ohlrogge JB (2006). Mitochondrial metabolism in developing embryos of *Brassica napus*. J Biol Chem.

[53] Xing S, van Deenen N, Magliano P, Frahm L, Forestier E, Nawrath C (2014). ATP citrate lyase activity is post-translationally regulated by sink strength and impacts the wax, cutin and rubber biosynthetic pathways. Plant J.

[54] Fatland BL, Nikolau BJ, Wurtele ES (2005). Reverse genetic characterization of cytosolic acetyl-CoA generation by ATP-citrate lyase in Arabidopsis. Plant Cell.

[55] Zell MB, Fahnenstich H, Maier A, Saigo M, Voznesenskaya EV, Edwards GE (2010). Analysis of Arabidopsis with highly reduced levels of malate and fumarate sheds light on the role of these organic acids as storage carbon molecules. Plant Physiol.

[56] Chia DW, Yoder TJ, Reiter W-D, Gibson SI (2000). Fumaric acid: An overlooked form of fixed carbon in Arabidopsis and other plant species. Planta.

[57] Dixon RA, Chen F, Guo D, Parvathi K (2001). The biosynthesis of monolignols: A “metabolic grid”, or independent pathways to guaiacyl and syringyl units?. Phytochemistry.

[58] Wang Y, Chantreau M, Sibout R, Hawkins S (2013). Plant cell wall lignification and monolignol metabolism. Front Plant Sci.

[59] Li P, Ponnala L, Gandotra N, Wang L, Si Y, Tausta SL (2010). The developmental dynamics of the maize leaf transcriptome. Nat Genet.

[60] Joshi V, Laubengayer KM, Schauer N, Fernie AR, Jander G (2006). Two Arabidopsis threonine aldolases are non-redundant and compete with threonine deaminase for a common substrate pool. Plant Cell.

[61] Masumoto C, Miyazawa S, Ohkawa H, Fukuda T, Taniguchi Y, Murayama S, Kusano M, Saito K, Fukayama H, Miyao M (2010). Phosphoenolpyruvate carboxylase intrinsically located in the chloroplast of rice plays a crucial role in ammonium assimilation. Proc Natl Acad Sci USA.

[62] Shi J, Keke Yi K, Liu Y, Xie L, Zhou Z, Chen Y, Hu Z, Zheng T, Liu R, Chen Y, Chen J (2015). Phosphoenolpyruvate carboxylase in Arabidopsis leaves plays a crucial role in carbon and nitrogen metabolism. Plant Physiol.

[63] Méret M, Kopetzki D, Degenkolbe T, Kleessen S, Nikoloski Z, Tellstroem V (2014). From systems biology to systems chemistry: Metabolomic procedures enable insight into complex chemical reaction networks in water. R Soc Chem Adv.

[64] Strehmel N, Kopka J, Scheel D, Böttcher C (2014). Annotating unknown components from GC/EI-MS-based metabolite profiling experiments using GC/APCI(+)-QTOFMS. Metabolomics.

[65] Bueschl C, Kluger B, Lemmens M, Adam G, Wiesenberger G, Maschietto V (2014). A novel stable isotope labelling assisted workflow for improved untargeted LC-HRMS based metabolomics research. Metabolomics.

[66] Murashige T, Skoog F (1962). A Revised medium for rapid growth and bio assays with tobacco tissue cultures. Physiol Plant.

[67] Schmidt S, Dethloff F, Beine-Golovchuk O, Kopka J (2013). The REIL1 and REIL2 proteins of *Arabidopsis thaliana* are required for leaf growth in the cold. Plant Physiol.

[68] Schneider CA, Rasband WS, Eliceiri KW (2012). NIH Image to ImageJ: 25 years of image analysis. Nat Methods.

[69] Team R (2015). RStudio: integrated development for R.

[70] Team RDC (2008). R: A language and environment for statistical computing.

[71] Saeed AI, Sharov V, White J, Li J, Liang W, Bhagabati N (2003). TM4: A free, open-source system for microarray data management and analysis. Biotechniques.

[72] Lisec J, Schauer N, Kopka J, Willmitzer L, Fernie AR (2006). Gas chromatography mass spectrometry-based metabolite profiling in plants. Nat Protoc.

[73] Wagner C, Sefkow M, Kopka J (2003). Construction and application of a mass spectral and retention time index database generated from plant GC/EI-TOF-MS metabolite profiles. Phytochemistry.

[74] Erban A, Schauer N, Fernie AR, Kopka J (2007). Non-supervised construction and application of mass spectral and retention time index libraries from time-of-flight gas chromatography-mass spectrometry metabolite profiles. Methods Mol Biol.

[75] Roessner U, Wagner C, Kopka J, Trethewey RN, Willmitzer L (2000). Simultaneous analysis of metabolites in potato tuber by gas chromatography–mass spectrometry. Plant J.

[76] Strehmel N, Hummel J, Erban A, Strassburg K, Kopka J (2008). Retention index thresholds for compound matching in GC-MS metabolite profiling. J Chromatogr B.

[77] Kopka J, Schauer N, Krüger S, Birkemeyer C, Usadel B, Bergmüller E (2005). GMD@CSB.DB: The Golm metabolome database. Bioinformatics.

[78] Schauer N, Steinhauser D, Strelkov S, Schomburg D, Allison G, Moritz T (2005). GC-MS libraries for the rapid identification of metabolites in complex biological samples. FEBS Lett.

[79] Hummel J, Strehmel N, Selbig J, Walther D, Kopka J (2010). Decision tree supported substructure prediction of metabolites from GC-MS profiles. Metabolomics.

[80] Wittmann C, Heinzle E (1999). Mass spectrometry for metabolic flux analysis. Biotechnol Bioeng.

[81] Van Winden WA, Wittmann C, Heinzle E, Heijnen JJ (2002). Correcting mass isotopomer distributions for naturally occurring isotopes. Biotechnol Bioeng.

[82] Huege J, Goetze J, Schwarz D, Bauwe H, Hagemann M, Kopka J (2011). Modulation of the major paths of carbon in photorespiratory mutants of *Synechocystis*. PLoS One.

