# Supplementary material for: Rapid in situ 13C tracing of sucrose utilization in Arabidopsis sink and source leaves
Source: Plant Methods. 2017 Oct 18;13:87. doi: 10.1186/s13007-017-0239-6 (PMC5648436; doi:10.1186/s13007-017-0239-6)
Supplement: Supplementary file 3 — Additional file 3: Figure S1. Examples of metabolites with pool size changes that are associated with the sink to source transition of leaves from an A. thaliana Col-0 rosette at vegetative growth stage 1.12–1.13. Normalized responses based on dry mass (mean ± standard error, n = 9–10). The mass spectra, retention indices, and updated annotations can be accessed through the Golm Metabolome Database (http://gmd.mpimp-golm.mpg.de/). [file 13007_2017_239_MOESM3_ESM.pptx]

## Slide 1
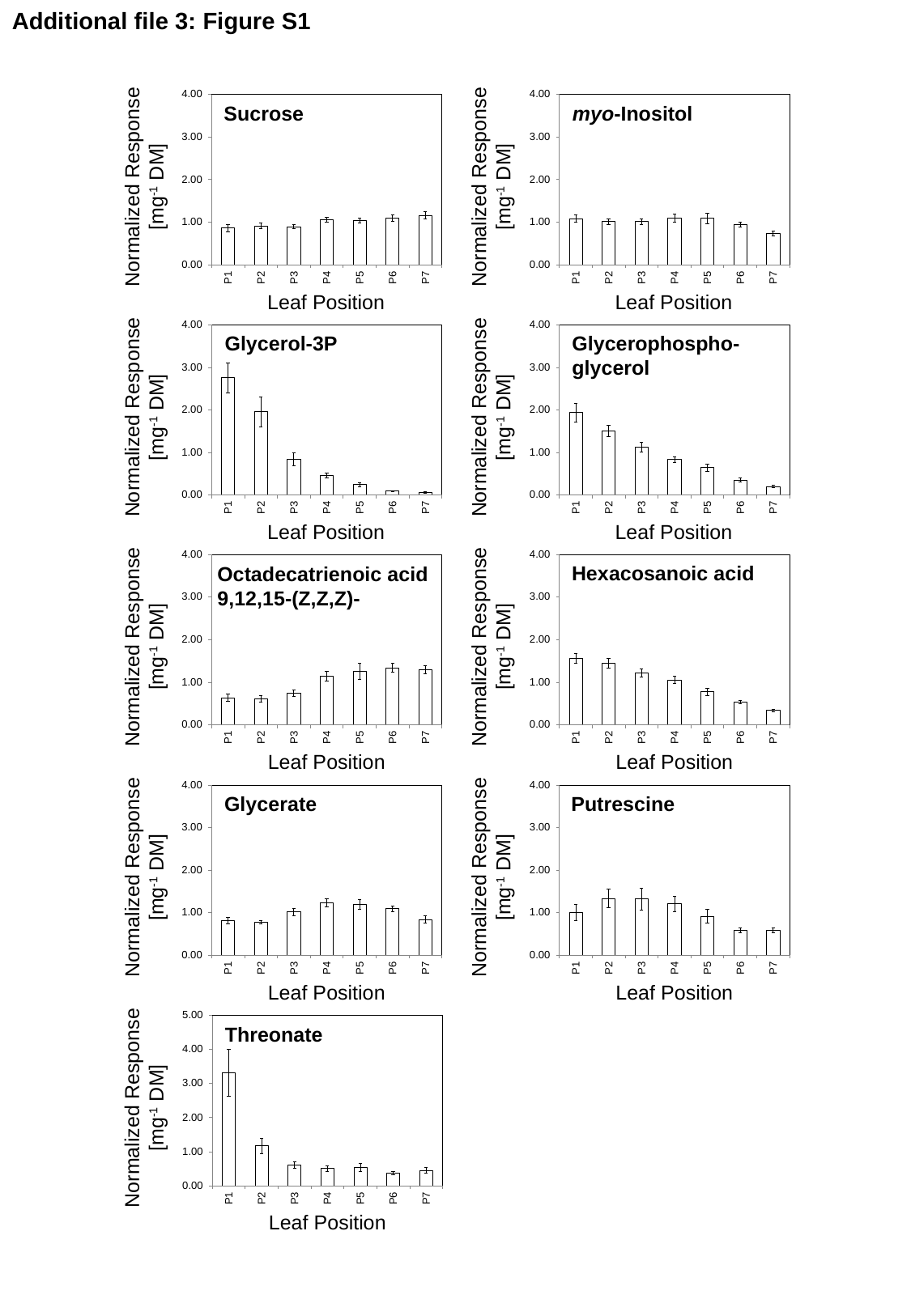

Additional file 3: Figure S1
Sucrose
myo-Inositol
Normalized Response
[mg-1 DM]
Normalized Response
[mg-1 DM]
Leaf Position
Leaf Position
Glycerol-3P
Glycerophospho-
glycerol
Normalized Response
[mg-1 DM]
Normalized Response
[mg-1 DM]
Leaf Position
Leaf Position
Hexacosanoic acid
Octadecatrienoic acid
9,12,15-(Z,Z,Z)-
Normalized Response
[mg-1 DM]
Normalized Response
[mg-1 DM]
Leaf Position
Leaf Position
Glycerate
Putrescine
Normalized Response
[mg-1 DM]
Normalized Response
[mg-1 DM]
Leaf Position
Leaf Position
Threonate
Normalized Response
[mg-1 DM]
Leaf Position
