# Supplementary figures and images for: Rapid in situ 13C tracing of sucrose utilization in Arabidopsis sink and source leaves
Source: Plant Methods. 2017 Oct 18;13:87. doi: 10.1186/s13007-017-0239-6 (PMC5648436; doi:10.1186/s13007-017-0239-6)

## Slide 1
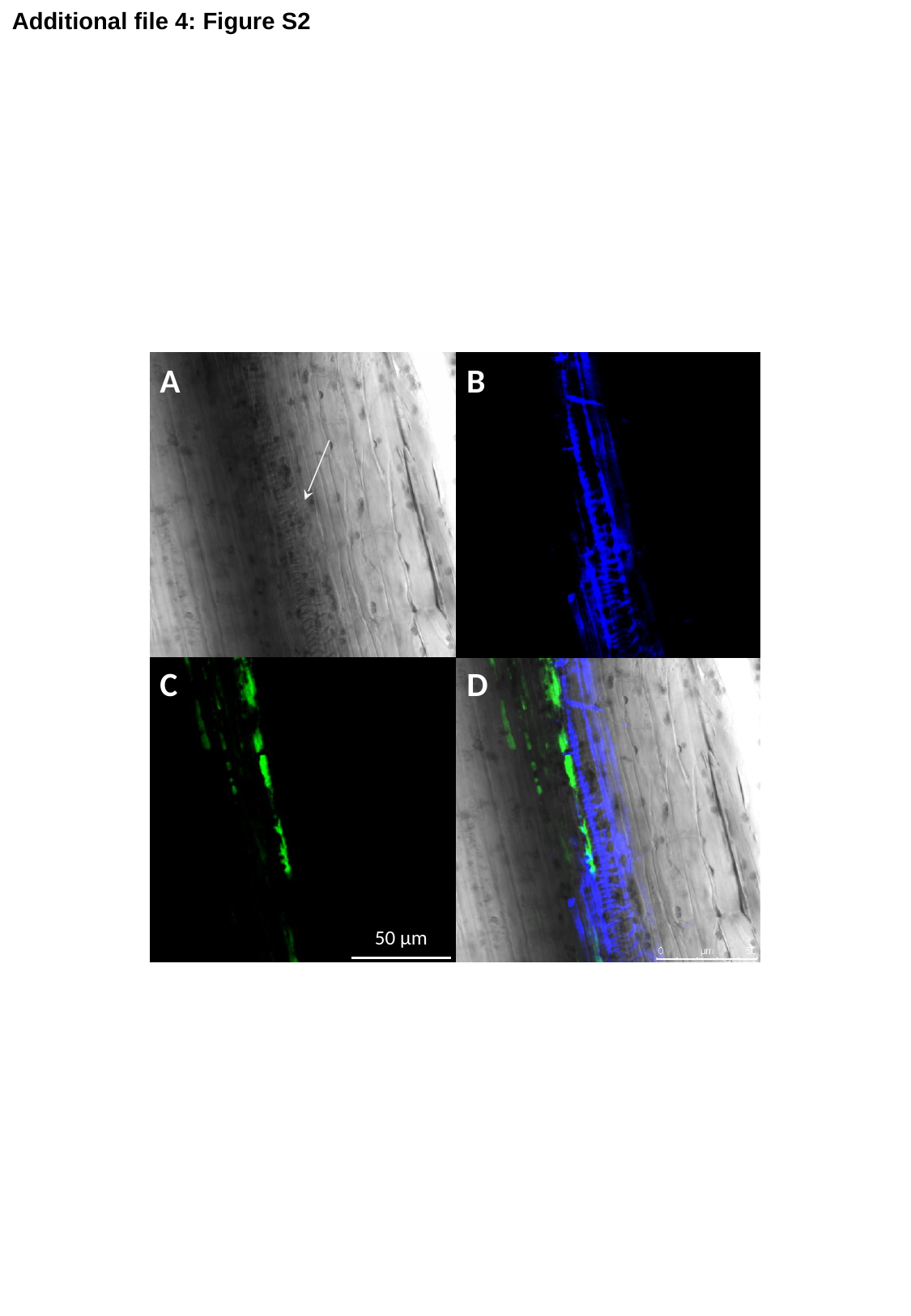

Additional file 4: Figure S2
50 µm
A
B
C
D

Supplement: Supplementary file 4 — Additional file 4: Figure S2. Simultaneous labeling of vascular tissue by co-feeding of 6-Carboxyfluorescein diacetate (green fluorescence) and Calcofluor White (blue fluorescence) dissolved in tap water. The fluorescent dyes were fed through the petiole of a transition leaf. A. thaliana plants were grown on soil under 8 h short day conditions and analysed at developmental stage 1.10–1.15. (A) Bright-field image of an approximately longitudinal optical section obtained by a confocal laser scanning microscope. The arrow indicates the position of a xylem vessel. (B) Phloem tissue indicated by 6-Carboxyfluorescein flourescence using excitation wave length λ = 488 nm and emission filter λ = 560 nm (green). (C) Xylem and apoplastic continuum indicated by Calcofluor White fluorescence using excitation wave length λ = 355 nm and an emission filter λ = 425 nm (blue). (D) Merged images (A–C). [file 13007_2017_239_MOESM4_ESM.pptx]
