# Supplementary material for: Rapid in situ 13C tracing of sucrose utilization in Arabidopsis sink and source leaves
Source: Plant Methods. 2017 Oct 18;13:87. doi: 10.1186/s13007-017-0239-6 (PMC5648436; doi:10.1186/s13007-017-0239-6)
Supplement: Supplementary file 6 — Additional file 6: Figure S3. Hierarchical clustering of A. thaliana leaf positions P2–P7 according to the 13C-labeling of metabolites 4 h after application of 13C sucrose using the HFA. Only metabolites that were labeled at all leaf positions were clustered using the Pearson’s correlation distance metric and complete linkage. Note that the labeling patterns of P2–P3 and P6–P7 were highly similar in contrast to the transition stage P4–P5. *Glutamine was determined as sum of glutamine and pyroglutamate [file 13007_2017_239_MOESM6_ESM.pptx]

## Slide 1
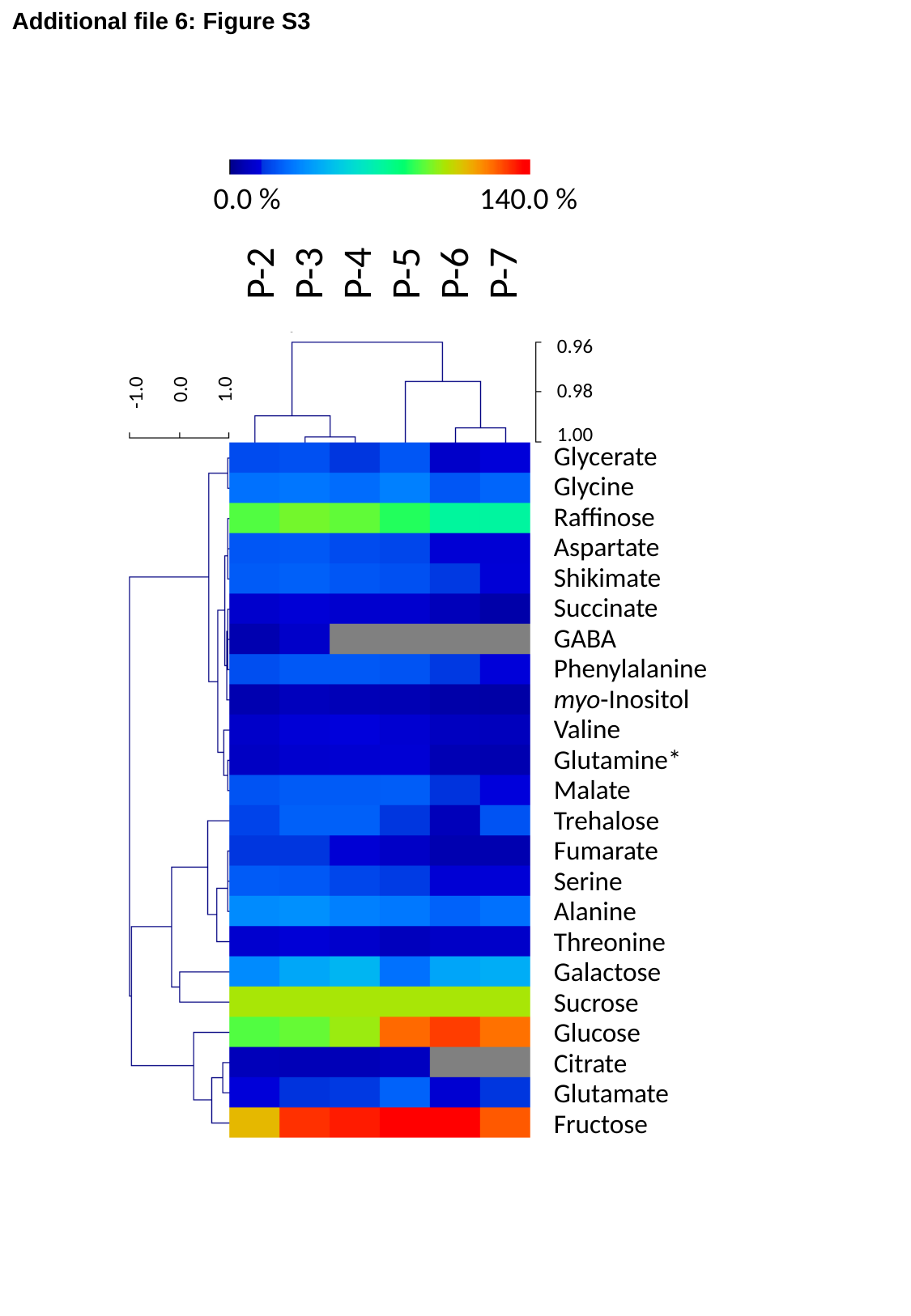

Additional file 6: Figure S3
P-2
P-3
P-4
P-5
P-6
P-7
0.0 % 140.0 %
0.96
0.98
1.00
-1.0
0.0
1.0
Glycerate
Glycine
Raffinose
Aspartate
Shikimate
Succinate
GABA
Phenylalanine
myo-Inositol
Valine
Glutamine*
Malate
Trehalose
Fumarate
Serine
Alanine
Threonine
Galactose
Sucrose
Glucose
Citrate
Glutamate
Fructose
