# Supplementary material for: Rapid in situ 13C tracing of sucrose utilization in Arabidopsis sink and source leaves
Source: Plant Methods. 2017 Oct 18;13:87. doi: 10.1186/s13007-017-0239-6 (PMC5648436; doi:10.1186/s13007-017-0239-6)
Supplement: Supplementary file 8 — Additional file 8: Figure S4. Qualitative analysis of mass isotopologue distributions (MIDs) of metabolites from the TCA cycle in sink leaves. Graphs show MIDs after correction for naturally occurring isotopes. Fragments were selected to represent MIDs of the complete carbon backbone as indicated below MIDs. The full MIDs of such fragments of aspartate and alanine were not detectable due to low fragment abundance. Instead we choose for comparison abundant fragments of aspartate and alanine that contained only part of the carbon backbone. For qualitative analysis we choose samples with typical low (blue) or medium (red) 13C enrichment that is indicated by inserts. Note that glutamate shows an increased abundance of M+2 (black arrow) that indicates preferred incorporation of 2 linked 13C atoms via acetyl-CoA. Malate and fumarate have enhanced M+3 (black arrows) relative to almost non-detectable M+4 that are consistent with preferred 12CO2 incorporation via the PEP carboxylase reaction. [file 13007_2017_239_MOESM8_ESM.pptx]

## Slide 1
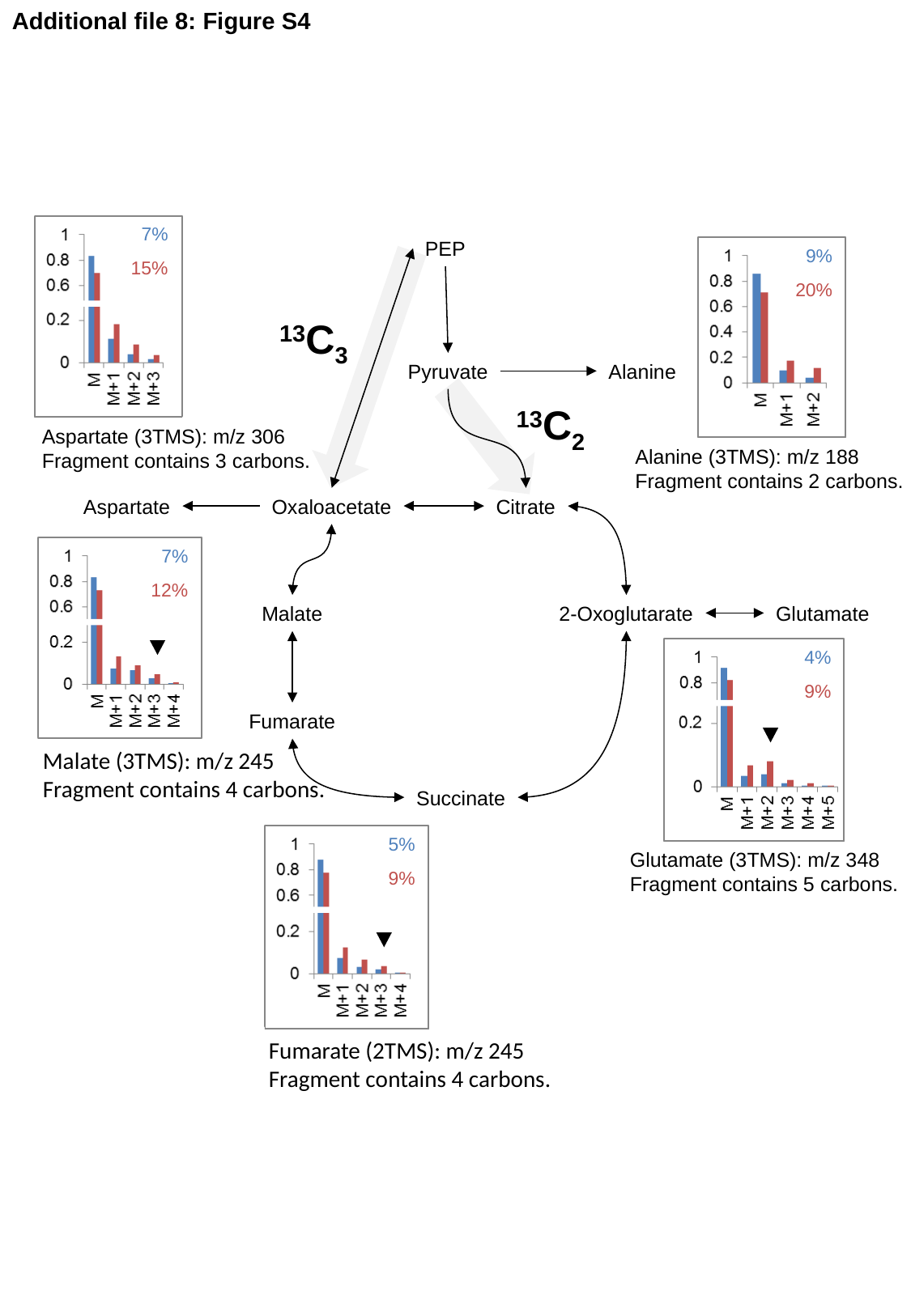

Additional file 8: Figure S4
7%
PEP
9%
15%
20%
13C3
Pyruvate
Alanine
13C2
Aspartate (3TMS): m/z 306
Fragment contains 3 carbons.
Alanine (3TMS): m/z 188
Fragment contains 2 carbons.
Aspartate
Oxaloacetate
Citrate
7%
12%
Malate
Glutamate
2-Oxoglutarate
4%
9%
Fumarate
Malate (3TMS): m/z 245
Fragment contains 4 carbons.
Succinate
5%
Glutamate (3TMS): m/z 348
Fragment contains 5 carbons.
9%
Fumarate (2TMS): m/z 245
Fragment contains 4 carbons.
